# Supplementary material for: Pandemic response in pluralistic health systems: a cross-sectional study of COVID-19 knowledge and practices among informal and formal primary care providers in Bihar, India
Source: BMJ Open. 2021 Apr 30;11(4):e047334. doi: 10.1136/bmjopen-2020-047334 (PMC8098292; doi:10.1136/bmjopen-2020-047334)
Supplement: Author's manuscript [file bmjopen-2020-047334.draft_revisions.pdf]

BMJ Open is committed to open peer review. As part of this commitment we make the peer review history of every article we publish publicly available.

When an article is published we post the peer reviewers' comments and the authors' responses online. We also post the versions of the paper that were used during peer review. These are the versions that the peer review comments apply to.

The versions of the paper that follow are the versions that were submitted during the peer review process. They are not the versions of record or the final published versions. They should not be cited or distributed as the published version of this manuscript.

BMJ Open is an open access journal and the full, final, typeset and author-corrected version of record of the manuscript is available on our site with no access controls, subscription charges or pay-per-view fees (http://bmjopen.bmj.com).

If you have any questions on BMJ Open's open peer review process please email info.bmjopen@bmj.com

BMJ Open

Embracing pluralism for more effective pandemic response: COVID-19 knowledge and practices of informal and formal primary care providers in India.

Journal:	*BMJ Open*
Manuscript ID	bmjopen-2020-047334
Article Type:	Original research
Date Submitted by the Author:	01-Dec-2020

Complete List of Authors:	Rao, Krishna; Johns Hopkins University Bloomberg School of Public Health, Department of International Health<br>kaur, Japneet<br>Peters, Michael<br>Kumar, Navneet; Oxford Policy Management<br>Nanda, Priya; Bill and Melinda Gates Foundation India

Keywords:	COVID-19, PRIMARY CARE, Quality in health care < HEALTH SERVICES ADMINISTRATION & MANAGEMENT

SCHOLARONE™ Manuscripts

I, the Submitting Author has the right to grant and does grant on behalf of all authors of the Work (as defined in the below author licence), an exclusive licence and/or a non-exclusive licence for contributions from authors who are: i) UK Crown employees; ii) where BMJ has agreed a CC-BY licence shall apply, and/or iii) in accordance with the terms applicable for US Federal Government officers or employees acting as part of their official duties; on a worldwide, perpetual, irrevocable, royalty-free basis to BMJ Publishing Group Ltd ("BMJ") its licensees and where the relevant Journal is co-owned by BMJ to the co-owners of the Journal, to publish the Work in this journal and any other BMJ products and to exploit all rights, as set out in our licence.

The Submitting Author accepts and understands that any supply made under these terms is made by BMJ to the Submitting Author unless you are acting as an employee on behalf of your employer or a postgraduate student of an affiliated institution which is paying any applicable article publishing charge ("APC") for Open Access articles. Where the Submitting Author wishes to make the Work available on an Open Access basis (and intends to pay the relevant APC), the terms of reuse of such Open Access shall be governed by a Creative Commons licence – details of these licences and which Creative Commons licence will apply to this Work are set out in our licence referred to above.

Other than as permitted in any relevant BMJ Author's Self Archiving Policies, I confirm this Work has not been accepted for publication elsewhere, is not being considered for publication elsewhere and does not duplicate material already published. I confirm all authors consent to publication of this Work and authorise the granting of this licence.

For peer review only

Embracing pluralism for effective pandemic response: COVID-19 knowledge and practices of informal and formal primary care providers in India.

Krishna D. Rao*, PhD, Johns Hopkins Bloomberg School of Public Health
Japneet Kaur, MA, Johns Hopkins Bloomberg School of Public Health
Michael A. Peters, MSPH, Johns Hopkins Bloomberg School of Public Health
Navneet Kumar, Oxford Policy Management
Priya Nanda, Bill and Melinda Gates Foundation

*Corresponding author: kdrao@jhu.edu

For peer review only - http://bmjopen.bmj.com/site/about/guidelines.xhtml

Embracing pluralism for effective pandemic response: COVID-19 knowledge and practices of informal and formal primary care providers in India.

Abstract

Background: In low and middle-income countries with pluralistic health systems primary care is typically provided by formal and informally trained health workers practicing several systems of medicine. In India, informal providers (IP) are usually first contact primary care providers for rural patients. As such, patients with COVID-19 symptoms will likely first visit an IP. This study assesses COVID-19 knowledge and management of suspect cases of IPs, trained practitioners of Indian systems of medicine (AYUSH), and allopathic medical doctors in India.

Methods: In a 2019 household survey we identified primary care providers who had served patients in the last month in the state of Bihar in eastern India. Providers were contacted via telephone in July 2020 when Bihar was experiencing a rapid increase in COVID cases. Using checklists we interviewed 452 IPs, 57 AYUSH practitioners, and 38 doctors (including 23 government doctors) from 224 villages across 34 of 38 districts in Bihar. The majority (96%) of sampled providers worked in a private health facility.

Findings: Most IPs and other private primary care providers reported no COVID-19 related engagement with government or civil society agencies. For them the principal source of COVID-19 information was television and newspapers. IP had reasonably good knowledge of typical COVID-19 symptoms and prevention, and at levels similar to medical doctors. However, there was low stated compliance among IPs (16%) and qualified primary care providers (15% of MBBS doctors, and 12% of AYUSH practitioners) with all WHO recommended management practices for suspect COVID-19 cases. Nearly half of IPs and other providers intended to treat COVID-19 suspects.

Interpretation: Poor management practices of COVID-19 suspects by rural primary care providers weakens government pandemic control efforts. Government action of providing information to IPs, as well as engaging then in contact tracing or public health messaging can strengthen pandemic control efforts.

Word count: 3,970

Strengths and limitations of this study

- This is the first large-scale survey to document informal provider (IP) and other private primary care provider COVID-19 knowledge, and case management practices in India.
- The study was conducted during the COVID-19 pandemic in the state of Bihar, India by contacting primary care providers by phone; as such, it provides insight into the practices of primary care providers during the pandemic.
- In LMICs like India which have a large presence of informal providers in the health workforce, rural COVID-19 suspects will likely first visit an informal provider first;

For peer review only - http://bmjopen.bmj.com/site/about/guidelines.xhtml

as such, study findings have important implications for pandemic control strategies in LMICs like India.

- This study is based on telephonic survey of primary care providers in Bihar and their stated practices may not fully reflect what they actually do in practice.

For peer review only

For peer review only - http://bmjopen.bmj.com/site/about/guidelines.xhtml

Introduction

Many low and middle-income countries have pluralistic health systems where clinical care providers practice several systems of medicine. This pluralism is further exacerbated by the presence of both formal and informally trained health workers, particularly in the primary care space. In India, informal providers (IP) are ubiquitous in the health workforce. IPs service a large share of primary care visits and are typically the first contact providers for patients seeking ambulatory care in rural or poor urban areas (1,2). As such, patients in these areas with common COVID-19 symptoms such as fever, fatigue, cough, or diarrhea, would likely first visit an IP. During disease outbreaks there is concern about how well health care providers are informed about the disease and its management, particularly when knowledge about the disease is rapidly changing. During previous outbreaks, such as SARS, MERS and Zika, collecting real-time information on provider knowledge and understanding of an emerging disease case management has supported response efforts by improving triage procedures and reducing infections in health care settings (3,4). Yet, despite the large presence of IPs in the primary care workforce little is known about their knowledge and practices related to COVID-19.

IPs have no formal medical training from a recognized institution, though they commonly have some form of informal training (5). In several countries, IPs are a substantial presence in the health workforce - they constitute around 88% of all health care providers in Bangladesh, and 77% in Uganda (5). India's pluralistic health system has a variety of formal and informal primary care providers – IPs, qualified AYUSH[1] physicians and allopathic doctors. IPs comprise between 24% to 43% of the health workforce in India, depending on how they are defined (6,7). IPs comprise a large share of the rural primary care health workforce - around 70% in rural compared to 31% of urban clinical care providers (7). Smaller scale state studies also indicate a large presence of IPs – for example one study found that in a district in the state of Karnataka 74% of the clinical care providers were IPs, while in a district in Uttarakhand, their share was 79% (1). People seek care from IPs for a number of reasons, including trust in the care IPs provide, proximity, and lower cost (8).

Indian IPs are a heterogenous group of medical providers who can practice allopathic, or Indian systems of medicines, or a mix of these(1). IPs typically treat common illnesses like fever, diarrhea, and respiratory conditions and play an important role in referring cases to higher-level health facilities(1). The few studies on IPs in India report a range of clinical experience – including certificate courses in allopathic and Indian systems of medicine, or apprenticeships with qualified clinicians (1). Studies on IP treatment practices find that they produce poor quality care, though they might be knowledgeable about treatment protocols (5). Interestingly, studies that have compared IPs with qualified primary care doctors find only small differences between them in protocol adherence, and no differences in the likelihood of giving a correct diagnosis or treatment (9).

Over the past year, several studies have assessed healthcare provider knowledge and perceptions of COVID-19. These studies have focused on qualified providers and report good knowledge of COVID-19, though there are important gaps to be addressed as best practices evolve over time (10). Studies among health providers in low- and middle-income

[1] AYUSH are trained practitioners of Indian systems of medicine and homeopathy i.e. Ayurveda, Yoga, Unani, Siddha and Homeopathy.

For peer review only

settings have echoed the broader literature by reporting good knowledge of COVID-19 symptoms and prevention, but gaps in case management protocols (11,12). In India, studies on the knowledge and practices of health care professionals such as doctors, medical residents, medical students, and other formally trained health workers reported high levels of knowledge of COVID-19 symptoms, and preventive measures, but do less well on case management (13–15). To the best of our knowledge, no study has attempted to document knowledge and practices related to COVID-19 among IPs. In general, studies on SARS and MERS reported that health care providers had good understanding of disease symptoms and prevention but did poorly on protocols for case management (16–19).

In this study we aim to understand the knowledge and practices of formal and informal primary care providers – IPs, AYUSH physicians, and allopathic doctors - related to COVID-19. Our study is based on a telephonic survey of primary care providers in the state of Bihar in eastern India. Understanding the knowledge and practices of IPs and other primary care providers in the context of the COVID-19 pandemic has important implications for the health care that communities receive, and more importantly, for the government's pandemic response.

Bihar (population 100 million) is among India's resource poor states. Although the state has made important gains in population health over the last few decades, it remains among the poorer performing states of India. Bihar's health system is under resourced, particularly for dealing with the COVID-19 outbreak(20). Its pluralistic health system is characterized by a large presence of informal providers. Our survey of primary care providers in the state indicated that 66% were IPs, 19% AYUSH physicians, 11% allopathic doctors, and 4% had other types of training (authors calculation based on 2020 provider survey). Further, around 68% of first contact primary care visits were to IPs, and around 59% of all primary care visits were to IPs (authors calculation based on 2020 household survey). At the time of this study in the first half of July 2020, Bihar (India) was experiencing a rapid increase in the number of COVID-29. Cases increased from around 1,000 (27,000) cases per day in mid-July to a high of 3,800 (90,000) in mid-September. The lockdown that was imposed across India in mid-March had severely affected the national economy and sent thousands of migrant workers back to Bihar from cities across India. The spread of COVID-19 in rural Bihar has been attributed to the return of migrant workers(21).

Methods

This paper is based on a cross sectional survey of primary health care providers in rural Bihar conducted via telephonic interviews. The survey was conducted from 1st to 15th July 2020, a period of rapid increase in the COVID-19 cases in the state.

Study sample: The primary care providers in this study were identified from a parent household survey conducted in Bihar between November 2019 and March 2020. This household survey was conducted to understand primary care seeking patterns. In this survey, 70 blocks across Bihar's nine divisions were selected using stratified systematic random sampling. Within each block, five villages were selected using PPS sampling. In each village, a probability sample of 30 households were selected using segmented random sampling. The household survey covered 70 blocks, 343 villages across all 38 districts in Bihar; a total of

For peer review only - http://bmjopen.bmj.com/site/about/guidelines.xhtml

8,356 households, and 39,477 individuals were sampled. Of the individuals sampled, 15,811 (40%) reported being ill in the past month, and 10,617 (71%) of them sought care outside their home.

Respondents who sought care outside home were asked to report details of the providers visited. We collected phone numbers and geolocations of these providers (if they were within 5 kilometers of the village) were collected with the idea of surveying these providers at a later date. Given the outbreak of coronavirus in March 2020 in India, the provider survey had to be suspended. However, we felt that we could contribute to the state's COVID-19 response by contacting these providers via telephone to understand their experiences during the outbreak. Any provider identified through the parent study were eligible for inclusion in the telephone survey.

A total of 9,497 provider contacts were recorded in the household survey. After cleaning to remove drug shops, duplicate providers, incomplete contact information we obtained telephone numbers of 1,138 private providers across 256 villages. Further we contacted a maximum of three providers per village, which reduced the target sample size to 1,103 providers. We made three attempts to contact respondents. Of the total of 1,103 providers contacted, we were able to successfully interview 522 private providers across 224 villages, achieving a 47% response rate. The key reasons for non-response included invalid telephone number (28%), respondent not interested in participating (19%), phone switched off (15%), and no response to calls (12%). At each block level Primary Health Center (PHC) in the 70 blocks covered by the household survey, the primary clinician was included in our study. Of the 70 PHCs contacted, we were able to conduct telephone interviews with from 25 PHC Medical Officers, which translates to a 36% response rate. Overall, our sample of providers covered 224 villages across 34 of 38 districts of Bihar.

Data collection: Providers were interviewed using a structured questionnaire to gather information on (i) change in patient care seeking, (ii) source of COVID-19 information, (iii) knowledge on COVID-19 spread, symptoms, and methods for prevention, and (iv) clinical management of COVID-19. Given the challenge of keeping the respondent engaged in a telephonic survey, every attempt was made to keep the tool short and precise. Average time taken to complete an interview was around 20 minutes. The phone surveys were carried out by Oxford Policy Management, Delhi. All enumerators possessed a nursing degree, had prior experience in conducting quantitative interviews, and were trained to conduct telephonic surveys using the COVID-19 knowledge tool. Data collected was recorded into an electronic data base using Computer-assisted telephonic interviewing (CATI) software to minimize information bias.

Data Analysis: Data was cleaned to identify incorrect entries. Variables of interest were mostly categorical and for most questions, respondents could select more than one response option. We conducted exploratory data analysis on the variables of interest to identify response patterns. Univariate and bivariate statistics were computed for variables of interest using two sample t-tests or chi-square tests of significance where necessary. Graphical analysis of the data was carried out. Missing data was not included in the analysis. Each provider was treated as an independent observation. Statistical analysis was conducted using Stata 14 (22).

For peer review only - http://bmjopen.bmj.com/site/about/guidelines.xhtml

Ethical approval: Ethical approval was granted by the Sigma Institutional Review Board in India (Reference number: 1007/IRB/20-21) as well as Johns Hopkins University Institutional Review Board. The purpose of the study was explained and oral informed consent was obtained from each respondent. If the respondent agreed to participate, then a signed copy of the consent form was sent to the respondent via SMS or WhatsApp.

Patient and public involvement: Questions for the household survey were pre-tested on the general public, including patients, and were appropriately modified. Reports of where sick household members sought treatment enabled identification and recruitment of primary care providers for this study. Further, the questionnaire was tailored so that it would not place an excessive burden on provider phone interviews.

Results

The 547 providers in our sample were from 224 villages across 34 of Bihar's 38 districts. Respondents were classified according to their self-reported qualification. A provider was classified as IP if they served as clinicians in a private facility and reported their training as any of the following - Registered Medical Practitioner (RMP), no formal qualification, diploma in modern and holistic medicine, nurse, pharmacist, community health worker, and a range of other non-degree qualifications. Providers who claimed to be trained in Indian systems of medicine were classified as AYUSH doctors, which is the official acronym for non-allopathic systems of medicine practiced in India. Providers who said they had a Bachelor of Medicine, Bachelor of Surgery (MBBS) or higher degree were classified as MBBS doctors. Note that all the providers in our sample had treated sick respondents in the parent household survey. Overall, there were 452 (83%) IPs, 57(10%) AYUSH providers, and 38 (7%) MBBS doctors in our sample. Of these, 522 (95%) were private and 25 (5%) were public providers. Private providers were distributed as follows - IPs: 452 (87%), AYUSH 55 (10%), and MBBS doctors 15 (3%). Among public sector providers there were 23 (92%) MBBS and 2 (8%) AYUSH doctors. All public providers were located either at a primary health care facility.

Providers were asked if they were still seeing patients despite the nation-wide lockdown. Among IPs, 73% reported seeing patients in person, 5% reported consulting patients over the phone, and 22% reported not seeing patients in the last week. Among MBBS and AYUSH doctors, 82% were seeing patients in person, 4% consulted patients over the phone, and 14% did not see patients in the last week. We asked providers who were still seeing patients if there was any change in the volume of patient visits the week before the survey as compared to what they usually experience (Figure 1). The vast majority of providers, irrespective of qualification or public or private sector, reported a fall in patient visits. Public sector providers most frequently reported an increase in patient visits.

[Figure 1 here]

A small (7%) proportion of private providers, including IPs, reported receiving training related to COVID-19 from either government or civil society sources, compared to 72% of public providers. Knowing where providers get their information on COVID-19 is important to plan future health communications activities (Table 1). Overall, there was no significant difference in sources of information across provider types, except for MBBS doctors.

For peer review only - http://bmjopen.bmj.com/site/about/guidelines.xhtml

Television was the most common source of information across provider types. For IPs and AYUSH providers, newspapers were the second most common source but for MBBS providers official government communications were most commonly cited knowledge sources after television. The difference seen in the MBBS group is due to the large number of public sector MBBS providers. The MBBS group comprises of 97% public and 3% private providers. Nearly 91% of the public MBBS providers reported receiving information from government sources, compared to 26% of private MBBS providers. Interestingly, mobile phones and the radio did not feature as important information sources.

[Table 1 here]

We asked providers to name common COVID-19 symptoms (Table 1). Overall, there was no significant differences in knowledge across provider types. The vast majority of providers in each group were able to identify symptoms such as fever, cough, and breathing problems. In contrast, much less known were symptoms such as loss of taste or smell and diarrhea, which are now seen as important and common symptoms of COVID-19 (23).

Common public health measures for preventing COVID-19 infection, such as using a facemask, washing hands and distancing from other people were widely known across provider types (Table 1). Others such as staying indoors or avoid touching one's face were much less reported. Overall, there was no significant difference across provider types in knowledge of COVID-19 prevention measures.

Of interest is to know how primary care providers in Bihar would manage a suspected case of COVID-19. We asked providers *"In the past week, if a patient came to you with fever, cough, and breathing difficulty, what would you tell them to do?"* According to the WHO ("Clinical Management of COVID-19: Interim guidance, May 27 2020") and Government of India (Clinical Management Protocol: COVID-19) guidelines, such a person would be a COVID-19 suspect having mild to moderate symptoms (23,24). We classified provider responses in terms of the WHO recommended actions for providers when presented with a suspected case of COVID-19 having mild to moderate symptoms (Figure 2). Overall, there was no significant difference between IPs and other provider types in following recommended actions, except for prescribing fever medication. A little more than half the primary care providers said they would require the patient to wear a mask during consultation. The majority said they would tell the patient to take a COVID-19 test. Testing advice was highest for IPs (69%), followed by AYUSH (63%), and MBBS doctors (63%). Prescribing fever medication to the patient varied significantly across provider types. Around 79% of MBBS doctors said they would prescribe fever medication, compared to around half the IPs and 39% of AYUSH providers. Advice about monitoring for complications was low across provider types – 44% of AYUSH, 41% of IPs, and 31% of MBBS doctors said they would ask the patient to check if the symptoms became worse after a few days. Assessing patients for risk factors of severe complications, such as existing health conditions of heart disease or diabetes, was reported by less than half the providers in any group – 44% AYUSH, 38% IPs and 31% MBBS doctors. There was no significant difference across provider types in assessing patients for risk factors associated with complications. Advising patients to isolate at home was recommended by around 60% of IPs and MBBS doctors, compared to 50% for AYUSH providers. Overall, only 16% of IPs, 15% of MBBS doctors, and 12% of AYUSH

For peer review only - http://bmjopen.bmj.com/site/about/guidelines.xhtml

practitioners reported all these recommended actions. On average, providers complied with 60% of the six recommended actions.

[Figure 2 here]

We asked providers if they would refer a patient who came to them in the past week with symptoms of fever, cough, and breathing difficulty to a higher level health facility (Figure 3). Nearly half the providers in each group said they would not refer such patients. Across provider types, among those who said they would refer, government clinics or hospital were the preferred places for referral (66% of referrals were to government clinics or hospitals). There was no significant difference across groups in referral patterns.

[Figure 3 here]

Discussion

Responding to pandemics is particularly challenging in pluralistic health systems. When diverse systems of medicine are practiced and there is a mix of informal and formal health workers, it is a challenge to achieve uniform standards in clinician understanding of the pandemic, ways to prevent infection and patient case management. This challenge is exacerbated by the ambiguous space that IPs occupy in India's health policy. One view, which reflects prevailing policy attitudes, is that IPs pose a danger to patients, and represent a problem that needs to be addressed. The alternative view is that they fill a vacuum in primary care service provision, and since they are already embedded within communities, it is pragmatic to engage with them. Findings from our study echo both perspectives. While IPs and other rural primary care providers were well informed about the basics of COVID-19 symptoms and preventive measures, they did poorly in terms of following recommended case management actions. On the other hand, as the COVID-19 pandemic spreads across rural India, IPs will likely to the first contact providers for many patients; as such, there is much to be gained if appropriate actions are taken by them in patient encounters. Further, because IPs are embedded in rural communities, they can play an important role in contact tracing, and in public health messaging.

One of the significant, though not unexpected, findings from this study was the little contact (or training) that IPs and other private primary care providers had with government or civil society agencies. One consequence of this is that the knowledge that most IPs and other primary care providers had on COVID-19 came from TV and newspapers. In contrast, most medical doctors in the government system reported receiving information on COVID-19 from government sources. Despite this, IPs and other primary care providers were remarkably well informed of certain COVID-19 symptoms and preventive measures. This echo findings from studies on the COVID-19 knowledge of qualified health professionals. Importantly, it highlights the importance of popular media sources in providing public health messaging to rural clinical providers. However, this may not be adequate. For example, providers had low awareness about diarrhea and the lack of taste or smell, which are now recognized as important COVID-19 symptoms. As such, there is a role for government in providing health information to primary care providers, particularly in the context of a pandemic.

For peer review only - http://bmjopen.bmj.com/site/about/guidelines.xhtml

Engaging private practitioners embedded in local communities has been an important strategy for controlling the COVID-19 pandemic. In the urban slum of Dharavi in Mumbai, one of the largest slum areas of the world, local government agencies have effectively controlled the COVID-19 outbreak using a range of measures, including using local health practitioners to engage their communities with public health messaging, screening, contact tracing, and providing clinical services (25). Community trust in local health care providers considerably aided government efforts in the pandemic response. In Bihar, and elsewhere in India, there have been earlier efforts by government and civil society organizations to engage with IPs and other private sector providers to improve quality of care (28). Yet, such actions have not been taken for the COVID-19 response. Clearly, providing information to IPs (and other private providers) on COVID-19, its prevention, and where testing centers are located can help improve community knowledge on preventive practices and management of suspected cases. Since IPs are embedded in local communities, they are an invaluable resource for contact tracing and transmitting public health messaging on COVID-19 to rural communities.

Our study finds that IPs and other formally trained providers had generally poor (stated) compliance with recommended case management practices for COVID-19. Their stated actions when presented with a COVID-19 suspect, such as – advising the patient to wear a mask, getting a COVID-19 test, prescribing fever medication, monitoring for complications, asking patients about risk factors, and advising isolation – were not universally practiced by IPs or even by formally trained providers. In fact, only a small minority of primary care providers stated that they would do all these recommended practices. This points to the poor COVID-19 case management practices among IPs and formally trained providers alike. The observation that IPs (or AYUSH providers) don't differ significantly from MBBS doctors in their stated practices confirms what has been reported in other studies (26). Findings from our study are likely generalizable to other rural contexts in resource limited states in India.

There are two notable limitations to our study. First, the high level of non-responses (55%), while common in telephone surveys, raises concerns about selection bias due to providers with certain characteristics not participating. There is some evidence for this – for example, among MBBS doctors there appears to be a higher non-response among public (75%) compared to private sector (46%) providers. If this non-response is related to the competency of respondents i.e. more competent doctors did not participate, then our estimates of compliance with quality actions would be biased downwards. However, studies report that in physician surveys, the extent of bias due to non-response is likely minimal because physicians are quite homogenous as a group in terms of knowledge and training, and variations that are present between them are unlikely to be associated with their likelihood of responding(27). A second concern relates to the assessment of COVID-19 case management; this is based on what respondents said they would do. For one, this could differ from what they do in practice. Studies that have examined differences in knowledge and practice have found significant gaps, particularly among more knowledgeable providers (28,29). While it is difficult to judge how well provider stated intentions are reflected in practice; however, their stated actions can be viewed as an upper limit of what they might do in practice. Further, in the context of a telephone interview, it is possible that there was underreporting of some key actions providers might take in practice because of trust issues. For example, it is somewhat surprising that so few IPs and AYUSH providers, who cannot officially prescribe allopathic medications, said they would prescribe fever medication (which is widely available) to someone with COVID-19 symptoms.

For peer review only - http://bmjopen.bmj.com/site/about/guidelines.xhtml

In pluralistic health systems where there is a large share of IPs and other private providers present in the health workforce, embracing all of them in the government's COVID-19 response offers several advantages. Because IPs and will likely be the first contact primary care provider for the majority of symptomatic individuals, how they might manage suspect cases becomes important to state and national efforts to control the outbreak. Moreover, because they are embedded within communities, IPs can assist in contact tracing, and public health messaging. As such, IPs can be an important partner in the government's COVID-19 response.

Author contribution: KDR, JK, MKP and PN were responsible for conceiving and designing the study. JK and NK were responsible for organizing data collection. KDR, JK, and MKP contributed to, data analysis. All authors contributed to writing and editing the manuscript.

Data sharing: Please contact Krishna D. Rao (kdrao@jhu.edu) for data related to this study.

Conflict of interest: None declared for KDR, JK, MKP, and NK. PN is employee of the funding agency.

Funding: Bill and Melinda Gates Foundation, Delhi. One of the authors (PN) is employed by the funder.

For peer review only - http://bmjopen.bmj.com/site/about/guidelines.xhtml

References

1. Gautham M, Shyamprasad KM, Singh R, Zachariah A, Singh R, Bloom G. Informal rural healthcare providers in North and South India. Health Policy Plan. 2014;29(SUPPL. 1):i20.
2. Priya R, Singh R, Das S. Health Implications of Diverse Visions of Urban Spaces: Bridging the Formal-Informal Divide. Front Public Heal. 2019 Sep;7:239.
3. Sharma S, Tyagi A, Ramachandra S, Bhuyan L, Dash K, Raghuvanshi M. Knowledge, attitudes, and practices among health-care providers regarding Zika virus infection. J Int Soc Prev Community Dent. 2018 Jan;8(1):41–7.
4. Deng JF, Olowokure B, Kaydos-Daniels SC, Chang HJ, Barwick RS, Lee ML, et al. Severe acute respiratory syndrome (SARS): Knowledge, attitudes, practices and sources of information among physicians answering a SARS fever hotline service. Public Health. 2006;120(1):15–9.
5. Sudhinaraset M, Ingram M, Lofthouse HK, Montagu D. What Is the Role of Informal Healthcare Providers in Developing Countries? A Systematic Review. PLoS One. 2013 Feb;8(2).
6. Karan A, Negandhi H, Nair R, Sharma A, Tiwari R, Zodpey S. Size, composition and distribution of human resource for health in India: New estimates using National Sample Survey and Registry data. BMJ Open. 2019 Apr;9(4):e025979.
7. Rao KD, Shahrawat R, Bhatnagar A. Composition and distribution of the health workforce in India: estimates based on data from the National Sample Survey. WHO South-East Asia J public Heal. 2016 Sep;5(2):133–40.
8. Anand G, Chhajed D, Shah S, Atkins S, Diwan V. Do qualifications matter? A qualitative study of how villagers decide their health care providers in a developing economy. Vermund SH, editor. PLoS One. 2019 Aug;14(8):e0220316.
9. Das J, Holla A, Das V, Mohanan M, Tabak D, Chan B. In urban and rural India, a standardized patient study showed low levels of provider training and huge quality gaps. Health Aff. 2012;31(12):2774–84.
10. Puspitasari IM, Yusuf L, Sinuraya RK, Abdulah R, Koyama H. Knowledge, Attitude, and Practice During the COVID-19 Pandemic: A Review. J Multidiscip Healthc. 2020 Jul;Volume 13:727–33.
11. Huynh G, Nguyen T, Tran V, Vo K, Vo V, Pham L. Knowledge and attitude toward COVID-19 among healthcare workers at District 2 Hospital, Ho Chi Minh City. Asian Pac J Trop Med. 2020 Jun;13(6):260–5.
12. Olum R, Chekwech G, Wekha G, Nassozi DR, Bongomin F. Coronavirus Disease-2019: Knowledge, Attitude, and Practices of Health Care Workers at Makerere University Teaching Hospitals, Uganda. Front Public Heal. 2020 Apr;8.
13. Mehrotra S, Jambunathan P, Jindal M, Gupta A, Kapoor K. A cross-sectional survey to assess the knowledge regarding coronavirus disease (COVID-19) among health care professionals. Med J Armed Forces India. 2020;
14. Kochuparambil J, Issac A, George S, Panicker N. Knowledge pertaining to COVID19 among medical population of Indian state of Kerala: An online cross-sectional survey. J Patient Saf Infect Control. 2020;8(1):17.
15. Kotian RP, Faujdar D, Kotian SP, D'souza B. Knowledge and understanding among medical imaging professionals in India during the rapid rise of the covid-19 pandemic. Health Technol (Berl). 2020 May;1.
16. Asaad A, El-Sokkary R, Alzamanan M, El-Shafei M. Knowledge and attitudes towards

For peer review only - http://bmjopen.bmj.com/site/about/guidelines.xhtml

middle east respiratory sydrome-coronavirus (MERS-CoV) among health care workers in South-Western Saudi Arabia. East Mediterr Heal J. 2020 Apr;26(4):435–42.
17. Khan MU, Shah S, Ahmad A, Fatokun O. Knowledge and attitude of healthcare workers about middle east respiratory syndrome in multispecialty hospitals of Qassim, Saudi Arabia. BMC Public Health. 2014;14(1).
18. Bener A, Al-Khal A. Knowledge, attitude and practice towards SARS. J R Soc Promot Health. 2004 Jul;124(4):167–70.
19. Tice AD, Kishimoto M, Dinh CH, Tak-Kin Lam G, Marineau M. Knowledge of Severe Acute Respiratory Syndrome among Community Physicians, Nurses, and Emergency Medical Responders. Prehosp Disaster Med. 2006 Jun;21(3):183–9.
20. Kumar A, Kumar M. Covid-19 and the public health system in Bihar. Econ Polit Wkly. 2020;
21. Mehrotra K. Over a month, Covid hits more districts, most in Bihar and UP. The Indian Express. 2020 May 22;
22. StataCorp. Stata Statistical Software Release 14. College Station, TX.; 2014.
23. Ministry of Health and Family Welfare. National Clinical Management Protocol: COVID-19 . Version 5. 2020.
24. World Health Organization. Clinical management of COVID-19. 2020.
25. Mohanan M, Babiarz KS, Goldhaber-Fiebert JD, Miller G, Vera-Hernández M. Effect of a large-scale social franchising and telemedicine program on childhood diarrhea and pneumonia outcomes in India. Health Aff. 2016;
26. Das J, Daniels B, Ashok M, Shim E-Y, Muralidharan K. Two Indias: The structure of primary health care markets in rural Indian villages with implications for policy. Soc Sci Med. 2020 Jun;112799.
27. Kellerman SE, Herold J. Physician response to surveys: A review of the literature. American Journal of Preventive Medicine. 2001.
28. Leonard KL, Masatu MC. The use of direct clinician observation and vignettes for health services quality evaluation in developing countries. 2005;61:1944–51.
29. Mohanan M, Vera-hernández M, Das V, Giardili S, Seth A. The Know-Do Gap in Quality of Health Care for Childhood Diarrhea and Pneumonia in Rural India. 2015;27708(4):349–57.

For peer review only

For peer review only - http://bmjopen.bmj.com/site/about/guidelines.xhtml

Figure 1: Percentage of providers reporting change in patient visits during COVID-19 outbreak

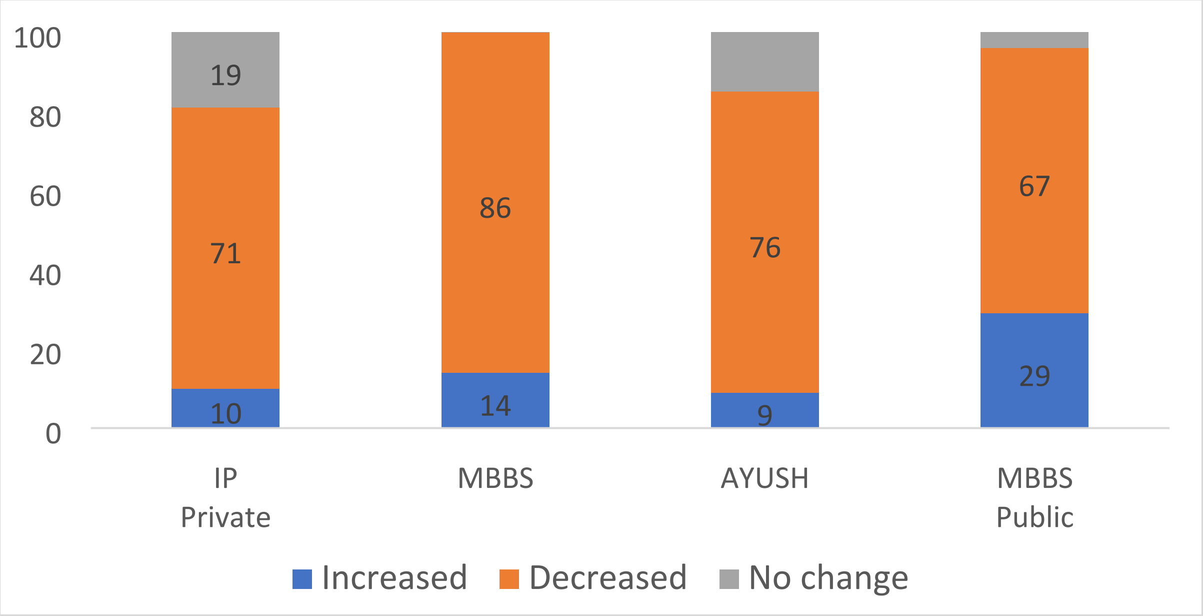

Figure 2: Provider stated compliance with WHO recommended actions for COVID-19 suspects

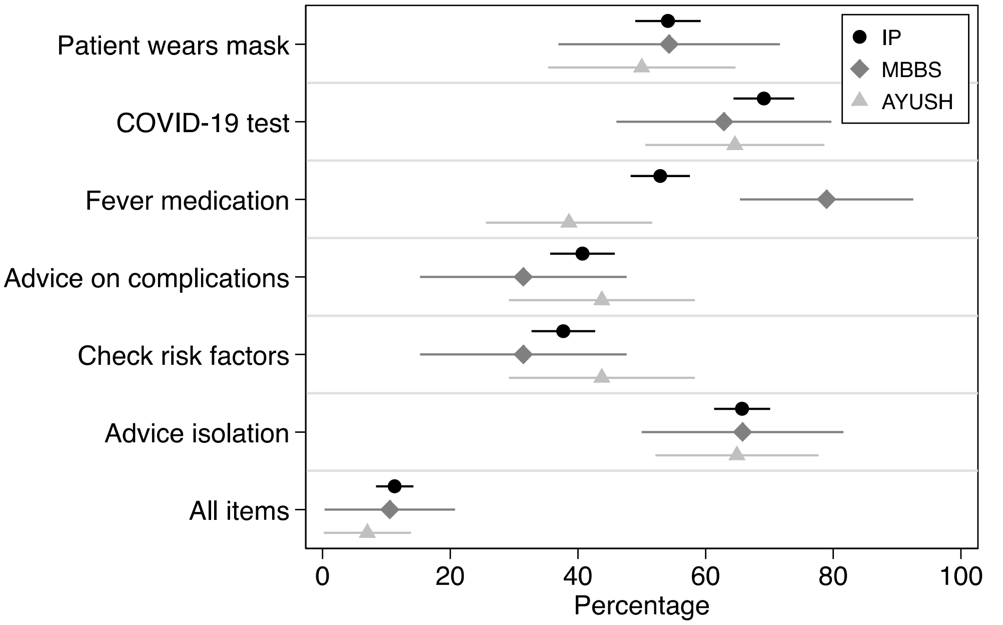

Note: (1) IP is Informal provider, MBBS is a provider with an MBBS degree, AYUSH is provider with a degree in Indian systems of medicine or homeopathy.
(2) Figures represent point estiamtes and 95% confidence intervals.
(3) Recommendations from Clinical management of COVID-19, Interim guidance, WHO, May 27, 2020.

For peer review only - http://bmjopen.bmj.com/site/about/guidelines.xhtml

Figure 3: Referral patterns for COVID-19 suspect cases

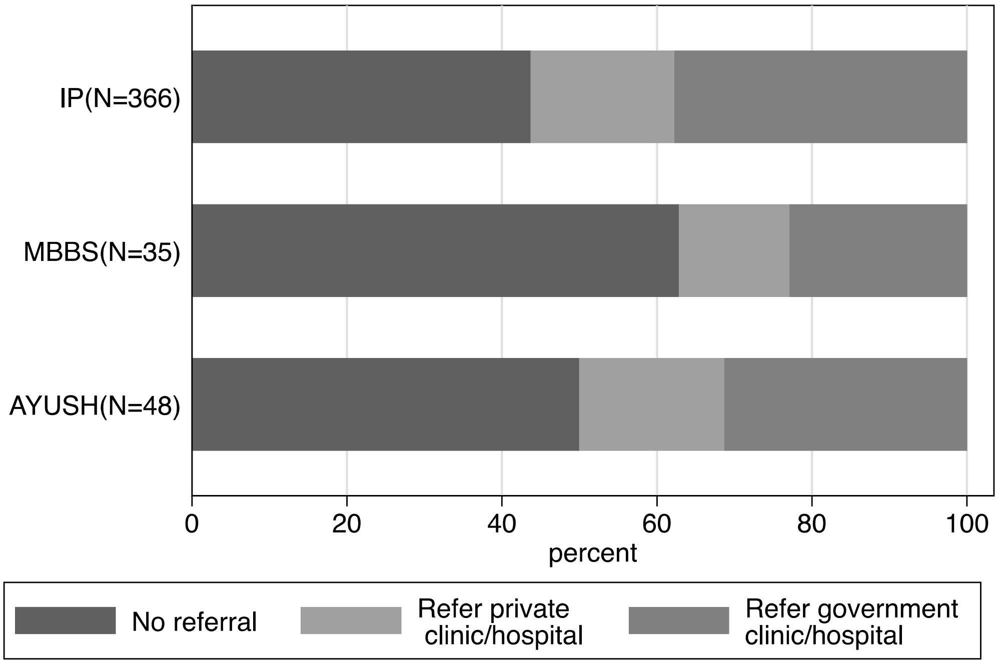

Note: Above resonses are to the question: In the past week if a patient came to you with symptoms of fever, cough, and breathing difficulty would you refer this patient to another doctor?

For peer review only

1

Table 1: Source of information and knowledge of COVID-19

	IP (%)	MBBS (%)	AYUSH (%)	Total (%)
**Sources of information**				
TV	76	63	74	75
Newspaper	53	42	40	51
Government	34	66	35	36*
Friends	24	11	23	23
Mobile phone	12	5	7	11
Radio	9	8	14	10
**Knowledge of COVID-19 symptoms**				
Fever	90	97	89	90
Cough	83	89	86	84
Breathing problem	71	76	79	72
Body ache	24	26	25	24
Sore throat	23	13	26	23
Fatigue	15	11	12	15
Diarrhea	6	13	9	7
Loss or taste/smell	8	24	5	9*
**Knowledge of COVID-19 prevention**				
Use facemask	83	89	79	83
Washing hands	80	89	74	80
Social distance	76	79	79	77
Stay at home	15	24	18	16
Avoid touching face	11	13	12	11
N(providers)	450	38	57	545

Note: (1) *Chi-sq test for group differences p-value < 0.05; (2) Respondents can select multiple responses.

For peer review only - http://bmjopen.bmj.com/site/about/guidelines.xhtml

Pandemic Response in Pluralistic Health Systems: A cross-sectional study of COVID-19 knowledge and practices among informal and formal primary care providers in Bihar, India.

Journal:	*BMJ Open*
Manuscript ID	bmjopen-2020-047334.R1
Article Type:	Original research
Date Submitted by the Author:	10-Feb-2021
Complete List of Authors:	Rao, Krishna; Johns Hopkins University Bloomberg School of Public Health, Department of International Health<br>kaur, Japneet; Johns Hopkins University<br>Peters, Michael; Johns Hopkins University<br>Kumar, Navneet; Oxford Policy Management<br>Nanda, Priya; Bill and Melinda Gates Foundation India
**Primary Subject Heading**:	Global health
Secondary Subject Heading:	Health policy, Health services research, Public health
Keywords:	COVID-19, PRIMARY CARE, Quality in health care < HEALTH SERVICES ADMINISTRATION & MANAGEMENT

SCHOLARONE™
Manuscripts

I, the Submitting Author has the right to grant and does grant on behalf of all authors of the Work (as defined in the below author licence), an exclusive licence and/or a non-exclusive licence for contributions from authors who are: i) UK Crown employees; ii) where BMJ has agreed a CC-BY licence shall apply, and/or iii) in accordance with the terms applicable for US Federal Government officers or employees acting as part of their official duties; on a worldwide, perpetual, irrevocable, royalty-free basis to BMJ Publishing Group Ltd ("BMJ") its licensees and where the relevant Journal is co-owned by BMJ to the co-owners of the Journal, to publish the Work in this journal and any other BMJ products and to exploit all rights, as set out in our licence.

The Submitting Author accepts and understands that any supply made under these terms is made by BMJ to the Submitting Author unless you are acting as an employee on behalf of your employer or a postgraduate student of an affiliated institution which is paying any applicable article publishing charge ("APC") for Open Access articles. Where the Submitting Author wishes to make the Work available on an Open Access basis (and intends to pay the relevant APC), the terms of reuse of such Open Access shall be governed by a Creative Commons licence – details of these licences and which Creative Commons licence will apply to this Work are set out in our licence referred to above.

Other than as permitted in any relevant BMJ Author's Self Archiving Policies, I confirm this Work has not been accepted for publication elsewhere, is not being considered for publication elsewhere and does not duplicate material already published. I confirm all authors consent to publication of this Work and authorise the granting of this licence.

For peer review only

Pandemic Response in Pluralistic Health Systems: A cross-sectional study of COVID-19 knowledge and practices among informal and formal primary care providers in Bihar, India.

Krishna D. Rao*, PhD, Johns Hopkins Bloomberg School of Public Health
Japneet Kaur, MA, Johns Hopkins Bloomberg School of Public Health
Michael A. Peters, MSPH, Johns Hopkins Bloomberg School of Public Health
Navneet Kumar, Oxford Policy Management
Priya Nanda, Bill and Melinda Gates Foundation

*Corresponding author: kdrao@jhu.edu

For peer review only - http://bmjopen.bmj.com/site/about/guidelines.xhtml

Pandemic Response in Pluralistic Health Systems: A cross-sectional study of COVID-19 knowledge and practices among informal and formal primary care providers in Bihar, India.

Abstract

Background: In low and middle-income countries with pluralistic health systems, primary care is typically provided by formal and informally trained health workers practicing several systems of medicine. In India, informal providers (IP) are usually first contact primary care providers for rural patients. As such, patients with COVID-19 symptoms will likely first visit an IP. This study assesses COVID-19 knowledge and case management of IPs, trained practitioners of Indian systems of medicine (AYUSH), and allopathic medical doctors in Bihar, India.

Methods: In a 2019 household survey we identified primary care providers who had served patients in the last month in the state of Bihar in eastern India. Providers were contacted via telephone in July 2020 when Bihar was experiencing a rapid increase in COVID-19 cases. Using checklists we interviewed 452 IPs, 57 AYUSH practitioners, and 38 doctors (including 23 government doctors) from 224 villages across 34 of 38 districts in Bihar. The majority (96%) of sampled providers worked in a private health facility.

Findings: Most IPs and other private primary care providers reported no COVID-19 related engagement with government or civil society agencies. For them, the principal source of COVID-19 information was television and newspapers. IPs had reasonably good knowledge of typical COVID-19 symptoms and prevention, and at levels similar to medical doctors. However, there was low stated compliance among IPs (16%) and qualified primary care providers (15% of MBBS doctors, and 12% of AYUSH practitioners) with all WHO recommended management practices for suspect COVID-19 cases. Nearly half of IPs and other providers intended to treat COVID-19 suspects without referral.

Interpretation: Poor management practices of COVID-19 suspects by rural primary care providers weakens government pandemic control efforts. Government action of providing information to IPs, as well as engaging them in contact tracing or public health messaging can strengthen pandemic control efforts.

Word count: 4,565

Strengths and limitations of this study

- This is the first large-scale survey to document informal providers' (IP) and other private primary care providers' COVID-19 knowledge, and case management practices in India.
- The study was conducted during the COVID-19 pandemic in the state of Bihar, India by contacting primary care providers by phone; as such, it provides insight into the practices of primary care providers during the pandemic.
- In LMICs like India which have a large presence of informal providers in the health workforce, rural COVID-19 suspects will likely first visit an informal provider; as

For peer review only

such, study findings have important implications for pandemic control strategies in LMICs like India.

- This study is based on telephonic survey of primary care providers in Bihar and their stated practices may not fully reflect what they actually do in practice.

For peer review only

Introduction

Many low- and middle-income countries (LMICs) have pluralistic health systems where clinical care providers practice several systems of medicine [1]–[3]. This pluralism is further exacerbated by the presence of both formal and informally trained health workers, particularly in the primary care space [3]. In India, informal providers (IP) are ubiquitous in the health workforce. IPs service a large share of primary care visits and are typically the first contact providers for patients seeking ambulatory care in rural or poor urban areas [4], [5]. As such, patients in these areas with common coronavirus disease (COVID-19) symptoms such as fever, fatigue, cough, or diarrhea, would likely first visit an IP. During disease outbreaks there is concern about how well health care providers are informed about the disease and its management, particularly when knowledge about the disease is rapidly changing. During previous outbreaks, such as Severe Acute Respiratory Syndrome (SARS) and Middle East Respiratory Syndrome (MERS), collecting real-time information on provider knowledge and understanding of an emerging disease case management has supported response efforts by improving triage procedures and reducing infections in health care settings [6]–[8]. Like SARS and MERS, COVID-19 is a coronavirus disease, spread by airborne transmission from close personal contact. Despite the large presence of IPs in the primary care workforce of many LMIC countries, little is known about their knowledge and practices related to COVID-19.

IPs have no formal medical training from a recognized institution, though they commonly have some form of informal training [3]. In several countries, IPs are a substantial presence in the health workforce - they constitute around 88% of all health care providers in Bangladesh, and 77% in Uganda [3]. In India most curative health services are provided by the private sector, though there is low health insurance coverage (about 13% of rural and 9% of urban population covered) and free care available at government clinics [9]. India's pluralistic health system has a variety of formal and informal primary care providers – IPs, qualified AYUSH[1] physicians, and allopathic doctors. IPs comprise between 24% to 43% of the health workforce in India, and their share of the health workforce varies greatly between and within states [10], [11]. IPs comprise a large share of the rural primary care health workforce - around 70% in rural compared to 31% of urban clinical care providers [11]. Smaller scale state studies also indicate a large presence of IPs – for example one study found that in a district in the state of Karnataka 74% of the clinical care providers were IPs, while in a district in Uttarakhand, their share was 79% [4], [11]. In general, IPs are trusted community members who practice within villages and charge fees-for-services which are paid for out-of-pocket. People seek care from IPs for a number of reasons, including trust in the care IPs provide, proximity, and lower cost relative to formally trained private providers [12].

Indian IPs are a heterogenous group of medical providers who can practice allopathic, or Indian systems of medicines, or a mix of these[4]. IPs typically treat common illnesses like fever, diarrhea, and respiratory conditions and play an important role in referring cases to higher-level health facilities [4]. The few studies on IPs in India report a range of clinical experience – including certificate courses in allopathic and Indian systems of medicine, or apprenticeships with qualified doctors [4]. Studies on IP treatment practices find that they produce poor quality care, though they might be knowledgeable about treatment protocols

[1] AYUSH are trained practitioners of Indian systems of medicine and homeopathy i.e. Ayurveda, Yoga, Unani, Siddha and Homeopathy.

For peer review only - http://bmjopen.bmj.com/site/about/guidelines.xhtml

[3]. Interestingly, studies that have compared IPs with qualified primary care doctors find only small differences between them in protocol adherence, and no differences in the likelihood of giving a correct diagnosis or treatment [13].

Over the past year, several studies have assessed healthcare provider knowledge and perceptions of COVID-19. These studies have focused on qualified providers and report good knowledge of COVID-19, though there are important gaps to be addressed as best practices evolve over time [14]. Studies among health providers in low- and middle-income settings have echoed the broader literature by reporting good knowledge of COVID-19 symptoms and prevention, but gaps in case management protocols [15], [16]. In India, studies on the knowledge and practices of health care professionals such as doctors, medical residents, medical students, and other formally trained health workers reported high levels of knowledge of COVID-19 symptoms, and preventive measures, but suggested lower levels of proficiency in terms of case management [17]–[19]. To the best of our knowledge, no study has attempted to document knowledge and practices related to COVID-19 among IPs. In general, studies on SARS and MERS reported that health care providers had good understanding of disease symptoms and prevention but did poorly on protocols for case management [20]–[23].

In this study we aim to understand the knowledge and practices of formal and informal primary care providers – IPs, AYUSH physicians, and allopathic doctors - related to COVID-19. Our study is based on a telephonic survey of primary care providers in the state of Bihar in eastern India. Understanding the knowledge and practices of IPs and other primary care providers in the context of the COVID-19 pandemic has important implications for the health care that communities receive, and more importantly, for the government's pandemic response.

Methods

This paper is based on a cross sectional survey of primary health care providers in rural Bihar conducted via telephonic interviews. The survey was conducted from 1st to 15th July 2020, a period of rapid increase in the COVID-19 cases in the state.

Setting: With a population of over 100 million and a GDP per capita of US $640, Bihar is among India's resource poor states. It's residents are spread across 38 districts and some 45,000 villages, 88% of which are considered to be located in rural areas [24]. Although the state has made important gains in population health over the last few decades, it remains among the poorer performing states of India. Bihar's health system is under resourced, including its human resources for health workforce, which is operating at 1.5 health workers per 10,000 population, well under WHO's recommended 22.8 workers per 10,000 population [11]. Bihar's pluralistic health system is characterized by a large presence of IPs, particularly in rural areas. This shortage of health workers and dependence on informal providers has hampered Bihar's ability to deal with the COVID-19 outbreak [25]. At the time of this study, in the first half of July 2020, Bihar was experiencing a rapid increase in the number of COVID-19 cases. Confirmed cases increased from around 400 cases per day at the beginning of July to about 1,300 cases per day by mid-July. Daily new cases continued to steadily

For peer review only - http://bmjopen.bmj.com/site/about/guidelines.xhtml

increase to a peak of 3,900 in mid-August. From the beginning of July to end of August, India as a whole experienced more than a three-fold increase in cases, from approximately 19,000 to 70,000 new cases per day. This came even after India instituted one of the strictest national lockdowns in the world which lasted from mid-March until the end of May. Under the lockdown, people were restricted from leaving their homes and all transport all transport services, educational institutions, and hospitality services were suspended- violators were punishable by up to a year in jail. The lockdown severely affected the national economy and forced thousands of migrant workers to return to Bihar from cities across India. The spread of COVID-19 in rural Bihar has in part been attributed to the return of these migrant workers [26].

Study sample: The primary care providers in this study were identified from a parent household survey conducted in Bihar between November 2019 and March 2020. This household survey was conducted to understand primary care seeking patterns. In this survey, 70 blocks (of 534 total blocks) across Bihar's nine divisions were selected using stratified systematic random sampling. Within each block, five villages were selected using probability proportional to size sampling. In each village, a probability sample of 30 households were selected using segmented random sampling. The household survey covered 70 blocks, 343 villages across all 38 districts in Bihar; a total of 8,356 households, and 39,477 individuals were sampled. Of the individuals sampled, 15,811 (40%) reported being ill in the past month, and 10,617 (71%) of them sought care outside their home.

Respondents who sought care outside home were asked to report details of the providers visited. We collected phone numbers and geolocations of these providers (if they were within 5 kilometers of the village) with the idea of surveying these providers at a later date. Given the outbreak of coronavirus in March 2020 in India, the provider survey had to be suspended. However, we felt that we could contribute to the state's COVID-19 response by contacting these providers via telephone to understand their experiences during the outbreak. Any provider identified through the parent study was eligible for inclusion in the telephone survey.

A total of 9,497 provider contacts were recorded in the household survey. Of these, we had complete contact information for 6,717 providers. After cleaning to remove drug shops (1,603), community health workers (35), and duplicate providers (3,941), we obtained telephone numbers of 1,138 private providers across 256 villages. We made three attempts to contact each respondent. Of the total of 1,138 providers contacted, we were able to successfully interview 522 private providers across 224 villages, achieving a 46% response rate. The key reasons for non-response included invalid telephone number (28%), respondent not interested in participating (19%), phone switched off (15%), and no response to calls (12%). At each block level Primary Health Center (PHC) in the 70 blocks covered by the household survey, the primary doctor was included in our study. Of the 70 PHCs contacted, we were able to conduct telephone interviews with from 25 PHC Medical Officers, which translates to a 36% response rate.

Data collection: Providers were interviewed using a structured questionnaire with choice-based answers to gather information on (i) change in patient care seeking, (ii) source of COVID-19 information, (iii) knowledge on COVID-19 spread, symptoms, and methods for prevention, and (iv) clinical management of COVID-19. Where provider answers were

For peer review only - http://bmjopen.bmj.com/site/about/guidelines.xhtml

ambiguous, enumerators were trained to probe the respondent to reach a clear answer, after which enumerators made a judgement on the most appropriate answer choice among the available selections. Given the challenge of keeping the respondent engaged in a telephonic survey, every attempt was made to keep the tool short and precise. Average time taken to complete an interview was around 20 minutes. The phone surveys were carried out by Oxford Policy Management, Delhi. All enumerators possessed a nursing degree, had prior experience in conducting quantitative interviews, and were trained to conduct telephonic surveys using computer-assisted telephonic interviewing (CATI) software integrated with CS Pro [27]. The CATI software displays the questionnaire on the screen of a tablet and the interviewer records the answers on the tablet during the interview. It also records the calls between the interviewer and provider enabling spot checks at a later date. Using CATI minimizes information bias as the skip logic is already embedded in the questionnaire and data is automatically recorded in a data management platform, removing the need for double data entry.

Data Analysis: A random subset of the calls recorded were checked by a data manager to identify any errors and mismatches with the data entered in CS Pro. Variables of interest were mostly categorical and for most questions, respondents could select more than one response option. Respondents were classified according to their self-reported medical training. A provider was classified as IP if they served as providers in a private facility and reported their training as any of the following - Registered Medical Practitioner (RMP), no formal qualification, diploma in modern and holistic medicine, nurse, pharmacist, community health worker, and a range of other non-degree qualifications. Providers who reported being trained in Indian systems of medicine were classified as AYUSH doctors. Providers who said they had a Bachelor of Medicine, Bachelor of Surgery (MBBS) or higher degree were classified as MBBS doctors. We conducted exploratory data analysis on the variables of interest to identify response patterns by provider types. Univariate and bivariate statistics were computed for variables of interest using two sample t-tests or chi-square tests of significance across provider types where necessary. Graphical analysis of the data was carried out. Missing data (responses from 2 IPs) was not included in the analysis. Each provider was treated as an independent observation. Statistical analysis was conducted using Stata 14 [28].

Ethical approval: Ethical approval was granted by the Sigma Institutional Review Board in India (Reference number: 1007/IRB/20-21) as well as Johns Hopkins University Institutional Review Board. The purpose of the study was explained and oral informed consent was obtained from each respondent. If the respondent agreed to participate, then a signed copy of the consent form was sent to the respondent via Short Message Service (SMS) text or WhatsApp.

Patient and public involvement: Questions for the household survey were pre-tested on the general public, including patients, and were appropriately modified. Reports of where sick household members sought treatment enabled identification and recruitment of primary care providers for this study. Further, the questionnaire was tailored so that it would not place an excessive burden on providers.

Results

The 547 providers included in our sample were from 224 villages across 34 of Bihar's 38 districts. Our sample included 452 (83%) IPs, 57 (10%) AYUSH providers, and 38 (7%)

For peer review only

For peer review only - http://bmjopen.bmj.com/site/about/guidelines.xhtml

MBBS doctors. Of these, 522 (95%) were private and 25 (5%) were public providers (Table 1). All public providers were located either at a primary health care facility or a community health center.

[Table 1 here]

Providers were asked if they were still seeing patients despite the nation-wide lockdown. Among IPs, 73% reported seeing patients in person, 5% reported consulting patients over the phone, and 22% reported not seeing patients in the last week. AYUSH doctors reported slightly higher rates of physical patient interactions, with 79% seeing patients, 4% consulting patients over the phone, and 18% not seeing any patients in the last week. Among MBBS doctors, 86% were seeing patients in person, 5% consulted patients over the phone, and 8% did not see patients in the last week. A higher percent of public MBBS doctors saw patients in the last week than private MBBS providers (91% vs 80%), and while a similar percent of MBBS providers did not see any patients in the last week (9% of public vs 7% of private), 13% of private MBBS doctors consulted patients over the phone, while no publicly employed MBBS doctors employed this technique. We asked providers who were still seeing patients if there was any change in the volume of patient visits the week before the survey as compared to what they usually experience (Figure 1). The vast majority of providers, irrespective of qualification or public or private sector, reported a fall in patient visits. However, nearly one fifth of IPs reported no change in patient volume over the prior week as compared to normal business. Public sector providers most frequently reported an increase in patient visits in the previous week.

[Figure 1 here]

A small (7%) proportion of private providers, including IPs, reported receiving training related to COVID-19 from either government or civil society sources, compared to 72% of public providers. Knowing where providers get their information on COVID-19 is important to plan future health communications activities (Table 2). Television was the most common source of information for all provider types except public MBBS doctors, for whom it was the second most common information source. For IPs, AYUSH providers, and private MBBS providers, newspapers were the second most common source of information. Nearly all (95%) of the public MBBS providers reported receiving information from government sources, compared to 29% of private MBBS providers, 34% of IPs, and 35% of AYUSH doctors. Interestingly, mobile phones and the radio did not feature as important information sources across provider types.

[Table 2 here]

We asked providers to name common COVID-19 symptoms (Table 2). Overall, there was no significant differences in knowledge across provider types. The vast majority of providers in each group were able to identify symptoms such as fever, cough, and breathing problems. In contrast, diarrhea was far less frequently identified as a symptom of COVID-19 across provider types. Interestingly, nearly a third of public MBBS providers identified loss of taste or smell as a COVID-19 symptom – a far greater percent than any other provider type.

For peer review only - http://bmjopen.bmj.com/site/about/guidelines.xhtml

Common public health measures for preventing COVID-19 infection, such as using a facemask, washing hands and distancing from other people were widely known across provider types (Table 2). Others such as staying indoors or avoid touching one's face were much less reported.

Of interest is to know how primary care providers in Bihar would manage a suspected case of COVID-19. We asked providers *"In the past week, if a patient came to you with fever, cough, and breathing difficulty, what would you tell them to do?"* According to the WHO ("Clinical Management of COVID-19: Interim guidance, May 27 2020") and Government of India (Clinical Management Protocol: COVID-19) guidelines, such a person would be a COVID-19 suspect having mild to moderate symptoms [29], [30]. We classified provider responses in terms of the WHO-recommended actions for providers when presented with a suspected case of COVID-19 having mild to moderate symptoms (Figure 2). The Government of India guidelines had substantial overlap with these WHO-recommended actions. Overall, there was no significant difference between IPs and other provider types in following recommended actions, except for prescribing fever medication. A little more than half the primary care providers said they would require the patient to wear a mask during consultation. The majority said they would tell the patient to take a COVID-19 test. Testing advice was highest for IPs (69%), followed by AYUSH (63%), and MBBS doctors (63%). Prescribing fever medication to the patient varied significantly across provider types. Around 79% of MBBS doctors said they would prescribe fever medication, compared to around half the IPs and 39% of AYUSH providers. Advice about monitoring for complications was low across provider types – 44% of AYUSH, 41% of IPs, and 31% of MBBS doctors said they would ask the patient to check if the symptoms became worse after a few days. Assessing patients for risk factors of severe complications, such as existing health conditions of heart disease or diabetes, was reported by less than half the providers in any group – 44% AYUSH, 38% IPs and 31% MBBS doctors. There was no significant difference across provider types in assessing patients for risk factors associated with complications. Advising patients to isolate at home was recommended by around 60% of IPs and MBBS doctors, compared to 50% for AYUSH providers. Overall, only 16% of IPs, 15% of MBBS doctors, and 12% of AYUSH practitioners reported all these recommended actions. On average, providers complied with 60% of the six recommended actions.

[Figure 2 here]

We asked providers if they would refer a patient who came to them in the past week with symptoms of fever, cough, and breathing difficulty to a higher level health facility (Figure 3). Nearly half the providers in each group said they would not refer such patients. Across provider types, among those who said they would refer, government clinics or hospital were the preferred places for referral (66% of referrals were to government clinics or hospitals). There was no significant difference across groups in referral patterns.

[Figure 3 here]

Discussion

Responding to pandemics is particularly challenging in pluralistic health systems. When diverse systems of medicine are practiced and there is a mix of informal and formal health

For peer review only - http://bmjopen.bmj.com/site/about/guidelines.xhtml

workers, it is a challenge to achieve uniform standards in providers' understanding of the pandemic, ways to prevent infection, and patient case management. This challenge is exacerbated by the ambiguous space that IPs occupy in India's health policy. One view, which reflects prevailing policy attitudes, is that IPs pose a danger to patients, and represent a problem that needs to be addressed. The alternative view is that they fill a vacuum in primary care service provision, and since they are already embedded within communities, it is pragmatic to engage with them. Findings from our study reflect both perspectives. Rural primary care providers as a whole were relatively well informed about the basics of COVID-19 symptoms and preventive measures, but performed poorly in terms of following recommended case management actions. In most cases, IPs performed similarly to MBBS or AYUSH doctors, but their low level of compliance could still endanger patients. On the other hand, over half of IPs recommended referring a suspect case to a government or other health clinic, so IPs could provide an important link to more sophisticated care. As the COVID-19 pandemic spreads across rural India, IPs will likely to the first contact providers for many patients; as such, there is much to be gained if appropriate actions are taken by them in patient encounters. Further, because IPs are embedded in rural communities, they can play an important role in contact tracing, and in public health messaging.

One of the significant, though not unexpected, findings from this study was the little contact (or training) that IPs and other private primary care providers had with government or civil society agencies. One consequence of this is that COVID-19 knowledge for most IPs and other primary care providers was primarily coming from TV and newspaper sources. In contrast, most medical doctors in the government system reported receiving information on COVID-19 from government sources. Despite this, IPs and other primary care providers were remarkably well informed of certain COVID-19 symptoms and preventive measures. This echoes findings from previous studies on the COVID-19 knowledge of qualified health professionals [15], [16]. Importantly, it highlights the importance and responsibility of popular media sources in providing public health messaging to rural clinical providers. However, this may not be adequate. For example, providers had low awareness about symptoms, especially diarrhea and the lack of taste or smell, which were described in the medical community as early as May 2020 and are now recognized as important COVID-19 symptoms [31]. Further, the importance of referral to clinics and testing sites could be further emphasized to better understand the local impact of the pandemic. While the frequency of referral between IPs and formal providers largely relies on established relationships and incentive structures, referral for COVID-19 testing could be an opportunity to strengthen linkages between the informal and formal sector [4]. As such, there is a role for government in providing health information to primary care providers, particularly in the context of a pandemic.

Engaging private practitioners embedded in local communities has been an important strategy for controlling the COVID-19 pandemic. In the urban slum of Dharavi in Mumbai, one of the largest slum areas of the world, local government agencies have effectively controlled the COVID-19 outbreak using a range of measures, including using local health practitioners to engage their communities with public health messaging, screening, contact tracing, and providing clinical services [32]. Community trust in local health care providers considerably aided government efforts in the pandemic response. In Bihar, and elsewhere in India, there have been earlier efforts by government and civil society organizations to engage with IPs and other private sector providers to improve quality of care [33]. Such actions have not yet

For peer review only - http://bmjopen.bmj.com/site/about/guidelines.xhtml

been taken for the COVID-19 response [34]. Clearly, providing information to IPs (and other private providers) on COVID-19, its prevention, and where testing centers are located can help improve community knowledge on preventive practices and management of suspected cases. Since IPs are embedded in local communities, they are an invaluable resource for contact tracing and sharing public health messaging on COVID-19 to rural communities.

Our study finds that rural primary care providers had generally poor (stated) compliance with recommended case management practices for COVID-19. Their stated actions when presented with a suspected COVID-19 case, such as advising the patient to wear a mask, getting a COVID-19 test, prescribing fever medication, monitoring the patient for complications, asking patients about risk factors, and advising isolation were generally not practiced by IPs and more importantly, not by formal providers, as the onus is on the formally trained. In fact, only a small minority of primary care providers stated that they would do all these recommended practices. This points to the poor COVID-19 case management practices among formally trained providers and IPs alike. AYUSH and MBBS doctors, from both public and private facilities, did not perform significantly better than IPs. The observation that IPs (or AYUSH providers) don't differ significantly from MBBS doctors in their stated practices confirms what has been reported in other studies [35]. Findings from our study are likely generalizable to other rural contexts in resource limited states in India.

There are two notable limitations to our study. First, the high level of non-responses (55%), while common in telephone surveys, raises concerns about selection bias due to providers with certain characteristics not participating. There is some evidence for this – for example, among MBBS doctors there appears to be a higher non-response among public (75%) compared to private sector (46%) providers. If this non-response is related to the competency of respondents i.e. more competent doctors did not participate, then our estimates of compliance with quality actions would be biased downwards. However, studies report that in physician surveys, the extent of bias due to non-response is likely minimal because physicians are quite homogenous as a group in terms of knowledge and training, and variations that are present between them are unlikely to be associated with their likelihood of responding [36]. A second concern relates to the assessment of COVID-19 case management; this is based on what respondents said they would do. For one, this could differ from what they do in practice. Studies that have examined differences in knowledge and practice have found significant gaps, particularly among more knowledgeable providers [37], [38]. While it is difficult to judge how well provider stated intentions are reflected in practice; however, their stated actions can be viewed as an upper limit of what they might do in practice. Further, in the context of a telephone interview, it is possible that there was underreporting of some key actions providers might take in practice because of trust issues. For example, it is somewhat surprising that so few IPs and AYUSH providers, who cannot officially prescribe allopathic medications, said they would prescribe fever medication (which is widely available) to someone with COVID-19 symptoms. While these providers could have answered that they prescribed other medicines such as cough medicines or antibiotics, about one fifth of all IPs and AYUSH providers answered that they would not prescribe any medicines.

In pluralistic health systems where there is a large share of IPs and other private providers present in the health workforce, embracing all of them in the government's COVID-19

For peer review only - http://bmjopen.bmj.com/site/about/guidelines.xhtml

response offers several advantages. Because IPs and will likely be the first contact primary care provider for the majority of symptomatic individuals, how they might manage suspect cases becomes vital to state and national efforts to control the outbreak. Moreover, because they are embedded within communities, IPs can assist in contact tracing, and public health messaging. As such, IPs can be an important partner in the government's COVID-19 response.

For peer review only

For peer review only - http://bmjopen.bmj.com/site/about/guidelines.xhtml

Author contribution: KDR, JK, MAP and PN were responsible for conceiving and designing the study. JK and NK were responsible for organizing data collection. KDR, JK, and MAP contributed to, data analysis. All authors contributed to writing and editing the manuscript.

Data sharing: Please contact Krishna D. Rao (kdrao@jhu.edu) for data related to this study.

Conflict of interest: None declared for KDR, JK, MKP, and NK. PN is employee of the funding agency.

Funding: Bill and Melinda Gates Foundation, Delhi. Grant # OPP119434. One of the authors (PN) is employed by the funder.

For peer review only

References

[1] B. Meessen *et al.*, "Composition of pluralistic health systems: how much can we learn from household surveys? An exploration in Cambodia," *Health Policy Plan.*, vol. 26, no. suppl_1, pp. i30–i44, Jul. 2011.

[2] G. Bloom and H. Standing, "Pluralism and marketisation in the health sector: meeting health needs in contexts of social change in low and middle-income countries," 2001.

[3] M. Sudhinaraset, M. Ingram, H. K. Lofthouse, and D. Montagu, "What Is the Role of Informal Healthcare Providers in Developing Countries? A Systematic Review," *PLoS One*, vol. 8, no. 2, Feb. 2013.

[4] M. Gautham, K. M. Shyamprasad, R. Singh, A. Zachariah, R. Singh, and G. Bloom, "Informal rural healthcare providers in North and South India," *Health Policy Plan.*, vol. 29, no. SUPPL. 1, p. i20, 2014.

[5] R. Priya, R. Singh, and S. Das, "Health Implications of Diverse Visions of Urban Spaces: Bridging the Formal-Informal Divide," *Front. Public Heal.*, vol. 7, p. 239, Sep. 2019.

[6] J. F. Deng *et al.*, "Severe acute respiratory syndrome (SARS): Knowledge, attitudes, practices and sources of information among physicians answering a SARS fever hotline service," *Public Health*, vol. 120, no. 1, pp. 15–19, 2006.

[7] A. J. Alsahafi and A. C. Cheng, "Knowledge, Attitudes and Behaviours of Healthcare Workers in the Kingdom of Saudi Arabia to MERS Coronavirus and Other Emerging Infectious Diseases," *Int. J. Environ. Res. Public Health*, vol. 13, no. 12, p. 1214, Dec. 2016.

[8] S. Al-Amri, R. Bharti, S. A. Alsaleem, H. M. Al-Musa, S. Chaudhary, and A. A. Al-Shaikh, "Knowledge and practices of primary health care physicians regarding updated guidelines of MERS-CoV infection in Abha city," *J. Fam. Med. Prim. care*, vol. 8, no. 2, pp. 455–461, Feb. 2019.

[9] Government of India, Ministry of Statistics & Programme Implementation, and National Statistical Office, "Health In India: National Sample Survey 75th Round," 2018.

[10] A. Karan, H. Negandhi, R. Nair, A. Sharma, R. Tiwari, and S. Zodpey, "Size, composition and distribution of human resource for health in India: New estimates using National Sample Survey and Registry data," *BMJ Open*, vol. 9, no. 4, p. e025979, Apr. 2019.

[11] K. D. Rao, R. Shahrawat, and A. Bhatnagar, "Composition and distribution of the health workforce in India: estimates based on data from the National Sample Survey," *WHO South-East Asia J. public Heal.*, vol. 5, no. 2, pp. 133–140, Sep. 2016.

[12] G. Anand, D. Chhajed, S. Shah, S. Atkins, and V. Diwan, "Do qualifications matter? A qualitative study of how villagers decide their health care providers in a developing economy," *PLoS One*, vol. 14, no. 8, p. e0220316, Aug. 2019.

[13] J. Das, A. Holla, V. Das, M. Mohanan, D. Tabak, and B. Chan, "In urban and rural India, a standardized patient study showed low levels of provider training and huge quality gaps," *Health Aff.*, vol. 31, no. 12, pp. 2774–2784, 2012.

[14] I. M. Puspitasari, L. Yusuf, R. K. Sinuraya, R. Abdulah, and H. Koyama, "Knowledge, Attitude, and Practice During the COVID-19 Pandemic: A Review," *J. Multidiscip. Healthc.*, vol. Volume 13, pp. 727–733, Jul. 2020.

[15] G. Huynh, T. Nguyen, V. Tran, K. Vo, V. Vo, and L. Pham, "Knowledge and attitude toward COVID-19 among healthcare workers at District 2 Hospital, Ho Chi Minh

For peer review only - http://bmjopen.bmj.com/site/about/guidelines.xhtml

City," *Asian Pac. J. Trop. Med.*, vol. 13, no. 6, pp. 260–265, Jun. 2020.
[16] R. Olum, G. Chekwech, G. Wekha, D. R. Nassozi, and F. Bongomin, "Coronavirus Disease-2019: Knowledge, Attitude, and Practices of Health Care Workers at Makerere University Teaching Hospitals, Uganda," *Front. Public Heal.*, vol. 8, Apr. 2020.
[17] S. Mehrotra, P. Jambunathan, M. Jindal, A. Gupta, and K. Kapoor, "A cross-sectional survey to assess the knowledge regarding coronavirus disease (COVID-19) among health care professionals," *Med. J. Armed Forces India*, 2020.
[18] J. Kochuparambil, A. Issac, S. George, and N. Panicker, "Knowledge pertaining to COVID19 among medical population of Indian state of Kerala: An online cross-sectional survey," *J. Patient Saf. Infect. Control*, vol. 8, no. 1, p. 17, 2020.
[19] R. P. Kotian, D. Faujdar, S. P. Kotian, and B. D'souza, "Knowledge and understanding among medical imaging professionals in India during the rapid rise of the covid-19 pandemic," *Health Technol. (Berl).*, p. 1, May 2020.
[20] A. Asaad, R. El-Sokkary, M. Alzamanan, and M. El-Shafei, "Knowledge and attitudes towards middle east respiratory sydrome-coronavirus (MERS-CoV) among health care workers in South-Western Saudi Arabia," *East. Mediterr. Heal. J.*, vol. 26, no. 4, pp. 435–442, Apr. 2020.
[21] M. U. Khan, S. Shah, A. Ahmad, and O. Fatokun, "Knowledge and attitude of healthcare workers about middle east respiratory syndrome in multispecialty hospitals of Qassim, Saudi Arabia," *BMC Public Health*, vol. 14, no. 1, 2014.
[22] A. Bener and A. Al-Khal, "Knowledge, attitude and practice towards SARS," *J. R. Soc. Promot. Health*, vol. 124, no. 4, pp. 167–170, Jul. 2004.
[23] A. D. Tice, M. Kishimoto, C. H. Dinh, G. Tak-Kin Lam, and M. Marineau, "Knowledge of Severe Acute Respiratory Syndrome among Community Physicians, Nurses, and Emergency Medical Responders," *Prehosp. Disaster Med.*, vol. 21, no. 3, pp. 183–189, Jun. 2006.
[24] Office of the Registrar General & Census Commissioner, "Census of India," 2011. [Online]. Available: http://www.censusindia.gov.in/2011census/hh-series/hh01.html. [Accessed: 03-Dec-2019].
[25] A. Kumar and M. Kumar, "Covid-19 and the public health system in Bihar," *Econ. Polit. Wkly.*, 2020.
[26] K. Mehrotra, "Over a month, Covid hits more districts, most in Bihar and UP," *The Indian Express*, New Delhi, May-2020.
[27] United States Census Bureau, "CS Pro," 2020. [Online]. Available: https://www.census.gov/data/software/cspro.html.
[28] StataCorp, "Stata Statistical Software Release 14," College Station, TX., 2014.
[29] Ministry of Health and Family Welfare, "National Clinical Management Protocol: COVID-19 ," *Version 5*, 03-Jul-2020. [Online]. Available: https://www.mohfw.gov.in/pdf/UpdatedClinicalManagementProtocolforCOVID19dated03072020.pdf. [Accessed: 20-Oct-2020].
[30] World Health Organization, "Clinical management of COVID-19," 27-May-2020. [Online]. Available: https://www.who.int/publications/i/item/clinical-management-of-covid-19. [Accessed: 20-Oct-2020].
[31] C. Menni *et al.*, "Real-time tracking of self-reported symptoms to predict potential COVID-19," *Nat. Med.*, vol. 26, no. 7, pp. 1037–1040, 2020.
[32] M. Golechha, "COVID-19 Containment in Asia's Largest Urban Slum Dharavi-Mumbai, India: Lessons for Policymakers Globally," *J. Urban Heal.*, vol. 97, no. 6,

For peer review only

pp. 796–801, 2020.
[33] M. Mohanan, K. S. Babiarz, J. D. Goldhaber-Fiebert, G. Miller, and M. Vera-Hernández, "Effect of a large-scale social franchising and telemedicine program on childhood diarrhea and pneumonia outcomes in India," *Health Aff.*, 2016.
[34] M. Gautham, G. Bloom, P. Balasubramaniam, C. Goodman, B. C. Forsberg, and A. Kumar, "Mobilising informal healthcare providers in India may help its response to covid-19," *The BMJ Opinion*, 2020. [Online]. Available: https://blogs.bmj.com/bmj/2020/06/24/mobilising-informal-healthcare-providers-in-india-may-help-its-response-to-covid-19/.
[35] J. Das, B. Daniels, M. Ashok, E.-Y. Shim, and K. Muralidharan, "Two Indias: The structure of primary health care markets in rural Indian villages with implications for policy," *Soc. Sci. Med.*, p. 112799, Jun. 2020.
[36] S. E. Kellerman and J. Herold, "Physician response to surveys: A review of the literature," *American Journal of Preventive Medicine*, vol. 20, no. 1. Elsevier Inc., pp. 61–67, 2001.
[37] K. L. Leonard and M. C. Masatu, "The use of direct clinician observation and vignettes for health services quality evaluation in developing countries," vol. 61, pp. 1944–1951, 2005.
[38] M. Mohanan, M. Vera-hernández, V. Das, S. Giardili, and A. Seth, "The Know-Do Gap in Quality of Health Care for Childhood Diarrhea and Pneumonia in Rural India," vol. 27708, no. 4, pp. 349–357, 2015.

For peer review only

Table 1: Sample characteristics

	Private Providers	Public Providers	Total providers
MBBS	15	23	38
AYUSH	55	2	57
Informal Provider	452	0	452
Total	522	25	547

Table 2: Source of information and knowledge of COVID-19

	IP (%)	Private MBBS (%)	Public MBBS (%)	AYUSH (%)	Total (%)
N(providers)	452	15	23	57	547
**Sources of information**					
TV	76	80	52	74	75
Newspaper	53	53	35	40	51
Government	34	27	91	35	36
Friends	24	20	4	23	23
Mobile phone	12	7	4	7	11
Radio	9	7	9	14	10
**Knowledge of COVID-19 symptoms**					
Fever	90	100	96	89	90
Cough	83	93	87	86	84
Breathing problem	71	67	83	79	72
Body ache	24	13	35	25	24
Sore throat	23	7	17	26	23
Fatigue	15	13	9	12	15
Diarrhea	6	13	13	9	7
Loss or taste/smell	8	13	30	5	9
**Knowledge of COVID-19 prevention**					
Use facemask	83	100	83	79	83
Washing hands	80	87	91	74	80
Social distance	76	67	87	79	77
Stay at home	15	33	17	18	16
Avoid touching face	11	7	17	12	11

Note: Respondents can select multiple responses. There were two missing values (both IPs) for sources of information, or knowledge of COVID-19 symptoms, or knowledge of COVID-19 prevention.

For peer review only - http://bmjopen.bmj.com/site/about/guidelines.xhtml

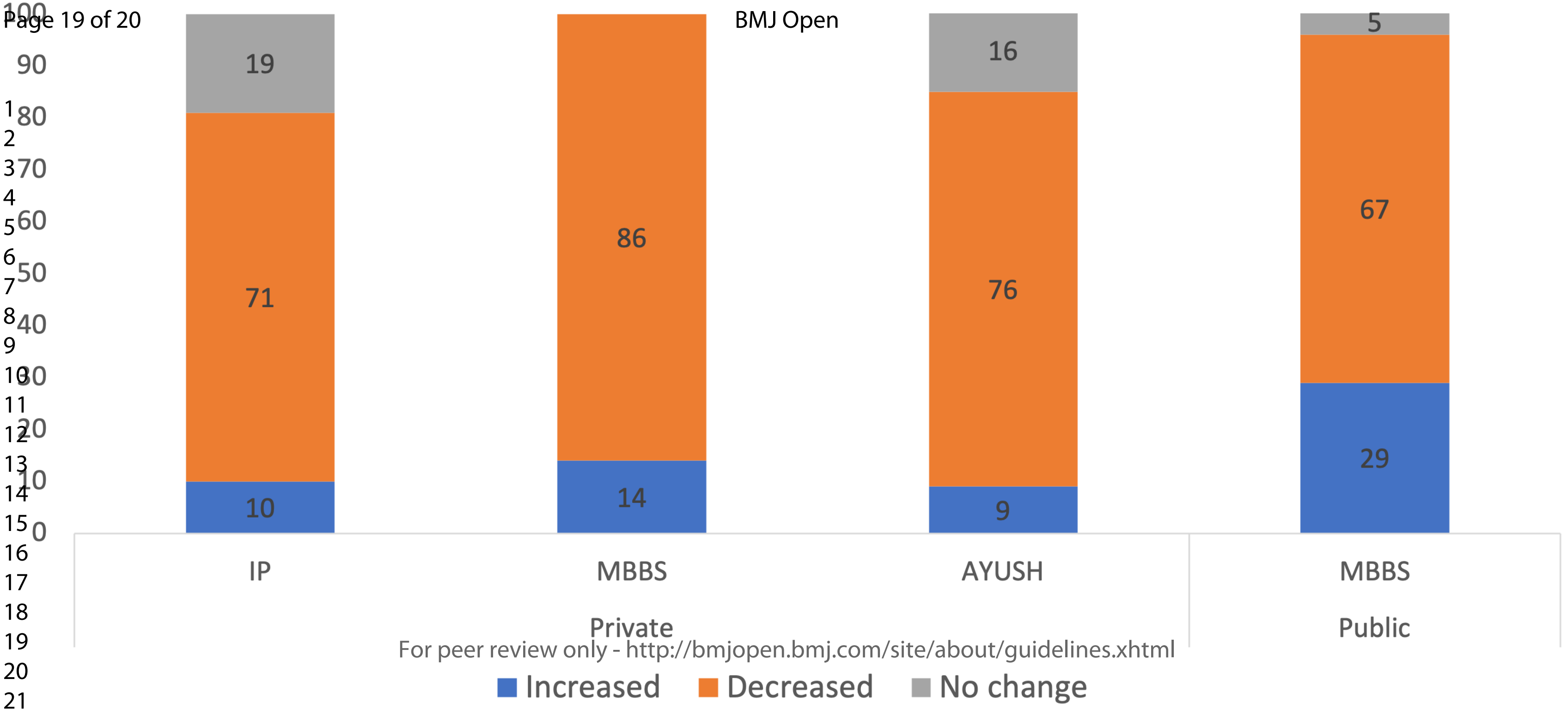
100
90
80
70
60
50
40
30
20
10
0
19
71
10
86
14
16
76
9
5
67
29
IP
MBBS
AYUSH
MBBS
Private
Public
Increased
Decreased
No change

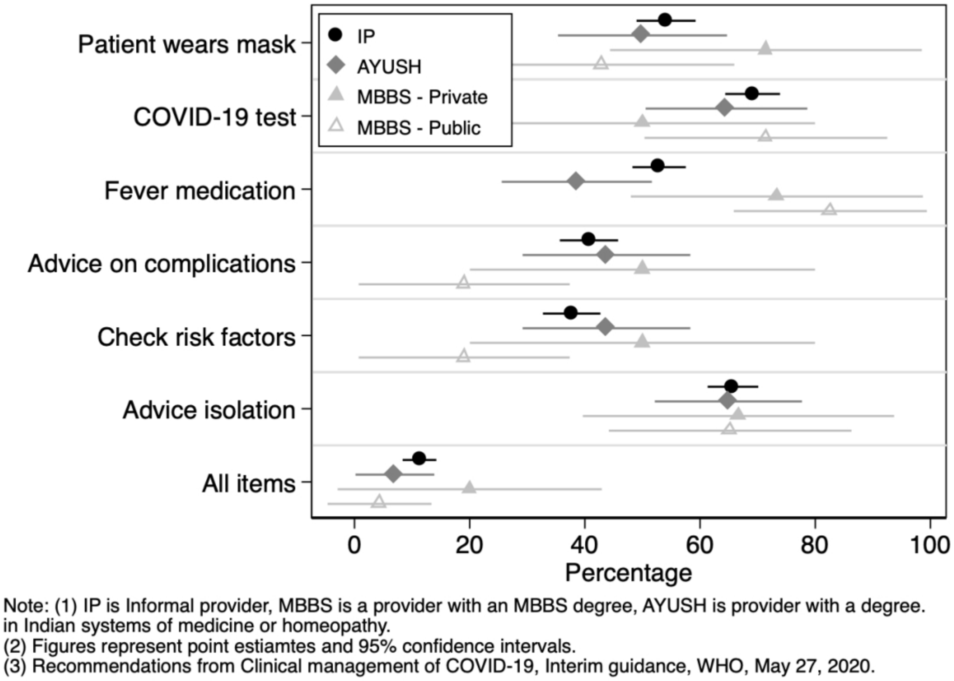

Figure 2: Provider stated compliance with WHO recommended actions for COVID-19 suspects

239x174mm (144 x 144 DPI)

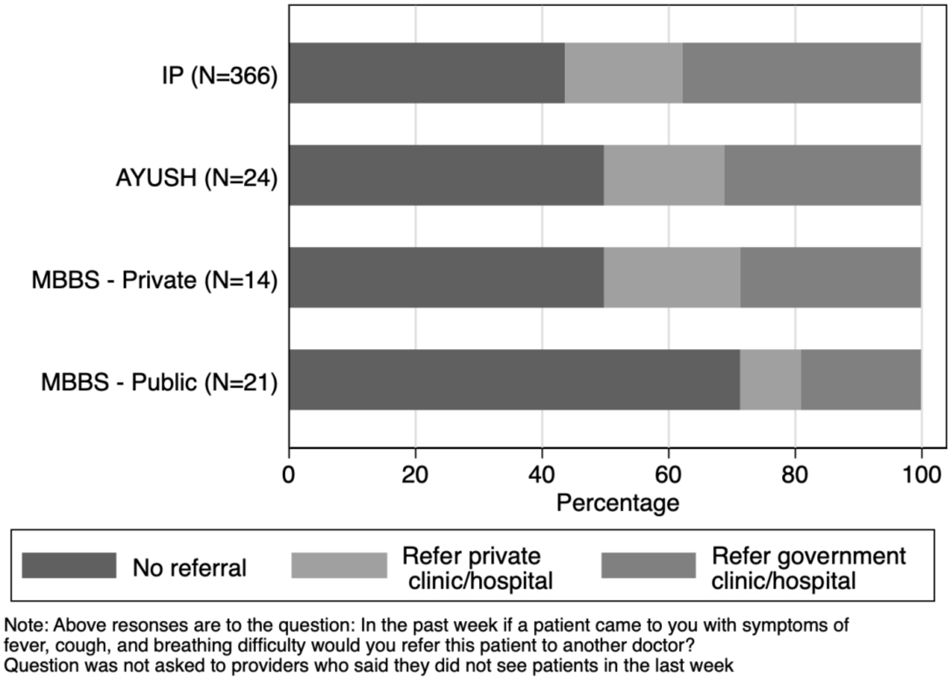

Figure 3: Referral patterns for COVID-19 suspect cases

261x190mm (144 x 144 DPI)

BMJ Open

Pandemic Response in Pluralistic Health Systems: A cross-sectional study of COVID-19 knowledge and practices among informal and formal primary care providers in Bihar, India.

Journal:	*BMJ Open*
Manuscript ID	bmjopen-2020-047334.R2
Article Type:	Original research
Date Submitted by the Author:	27-Mar-2021

Complete List of Authors:	Rao, Krishna; Johns Hopkins University Bloomberg School of Public Health, Department of International Health<br>kaur, Japneet; Johns Hopkins University<br>Peters, Michael; Johns Hopkins University<br>Kumar, Navneet; Oxford Policy Management<br>Nanda, Priya; Bill and Melinda Gates Foundation India

**Primary Subject Heading**:	Global health
Secondary Subject Heading:	Health policy, Health services research, Public health
Keywords:	COVID-19, PRIMARY CARE, Quality in health care < HEALTH SERVICES ADMINISTRATION & MANAGEMENT

SCHOLARONE™ Manuscripts

I, the Submitting Author has the right to grant and does grant on behalf of all authors of the Work (as defined in the below author licence), an exclusive licence and/or a non-exclusive licence for contributions from authors who are: i) UK Crown employees; ii) where BMJ has agreed a CC-BY licence shall apply, and/or iii) in accordance with the terms applicable for US Federal Government officers or employees acting as part of their official duties; on a worldwide, perpetual, irrevocable, royalty-free basis to BMJ Publishing Group Ltd ("BMJ") its licensees and where the relevant Journal is co-owned by BMJ to the co-owners of the Journal, to publish the Work in this journal and any other BMJ products and to exploit all rights, as set out in our licence.

The Submitting Author accepts and understands that any supply made under these terms is made by BMJ to the Submitting Author unless you are acting as an employee on behalf of your employer or a postgraduate student of an affiliated institution which is paying any applicable article publishing charge ("APC") for Open Access articles. Where the Submitting Author wishes to make the Work available on an Open Access basis (and intends to pay the relevant APC), the terms of reuse of such Open Access shall be governed by a Creative Commons licence – details of these licences and which Creative Commons licence will apply to this Work are set out in our licence referred to above.

Other than as permitted in any relevant BMJ Author's Self Archiving Policies, I confirm this Work has not been accepted for publication elsewhere, is not being considered for publication elsewhere and does not duplicate material already published. I confirm all authors consent to publication of this Work and authorise the granting of this licence.

For peer review only

Pandemic Response in Pluralistic Health Systems: A cross-sectional study of COVID-19 knowledge and practices among informal and formal primary care providers in Bihar, India.

Krishna D. Rao*, PhD, Johns Hopkins Bloomberg School of Public Health
Japneet Kaur, MA, Johns Hopkins Bloomberg School of Public Health
Michael A. Peters, MSPH, Johns Hopkins Bloomberg School of Public Health
Navneet Kumar, Oxford Policy Management
Priya Nanda, Bill and Melinda Gates Foundation

*Corresponding author: kdrao@jhu.edu

For peer review only

For peer review only - http://bmjopen.bmj.com/site/about/guidelines.xhtml

Pandemic Response in Pluralistic Health Systems: A cross-sectional study of COVID-19 knowledge and practices among informal and formal primary care providers in Bihar, India.

Abstract

Objectives:
This study assesses COVID-19 knowledge and case management of informal providers (IPs), trained practitioners of Ayurveda, Yoga and Naturopathy, Unani, Siddha and Homeopathy (AYUSH), and MBBS medical doctors providing primary care services in rural Bihar, India.

Design:
This was a cross-sectional study of primary care providers conducted via telephone between 1 and 15 July, 2020.

Setting:
Primary care providers from 224 villages in 34 districts across Bihar, India.

Participants:
452 IPs, 57 AYUSH practitioners, and 38 doctors (including 23 government doctors) were interviewed from a census of 1,138 primary care providers utilized by community members that could be reached by telephone.

Primary outcome measure(s):
Providers were interviewed using a structured questionnaire with choice-based answers to gather information on (i) change in patient care seeking, (ii) source of COVID-19 information, (iii) knowledge on COVID-19 spread, symptoms, and methods for prevention, and (iv) clinical management of COVID-19.

Results:
During the early days of the COVID-19 pandemic, 72% of providers reported a decrease in patient visits. Most IPs and other private primary care providers reported receiving no COVID-19 related engagement with government or civil society agencies. For them, the principal source of COVID-19 information was television and newspapers. IPs had reasonably good knowledge of typical COVID-19 symptoms and prevention, and at levels similar to doctors. However, there was low stated compliance among IPs (16%) and qualified primary care providers (15% of MBBS doctors, and 12% of AYUSH practitioners) with all WHO recommended management practices for suspect COVID-19 cases. Nearly half of IPs and other providers intended to treat COVID-19 suspects without referral.

Conclusions:
Poor management practices of COVID-19 suspects by rural primary care providers weakens government pandemic control efforts. Government action of providing information to IPs, as well as engaging them in contact tracing or public health messaging can strengthen pandemic control efforts.

Word count: 4,586

For peer review only - http://bmjopen.bmj.com/site/about/guidelines.xhtml

Strengths and limitations of this study

- This is the first large-scale survey to document informal providers' (IP) and other private primary care providers' COVID-19 knowledge, and case management practices in India.
- The study was conducted during the COVID-19 pandemic in the state of Bihar, India by contacting primary care providers by phone; as such, it provides insight into the practices of primary care providers during the pandemic.
- In LMICs like India which have a large presence of informal providers in the health workforce, rural COVID-19 suspects will likely first visit an informal provider; as such, study findings have important implications for pandemic control strategies in LMICs.
- This study is based on telephonic survey of primary care providers in Bihar and their stated practices may not fully reflect what they actually do in practice.

For peer review only

For peer review only - http://bmjopen.bmj.com/site/about/guidelines.xhtml

Introduction

Many low- and middle-income countries (LMICs) have pluralistic health systems where clinical care providers practice several systems of medicine [1]–[3]. This pluralism is further exacerbated by the presence of both formal and informally trained health workers, particularly in the primary care space [3]. In India, informal providers (IPs) are ubiquitous in the health workforce. IPs service a large share of outpatient visits and are typically the first contact providers for patients seeking ambulatory care in rural or poor urban areas [4], [5]. As such, patients in these areas with common coronavirus disease (COVID-19) symptoms such as fever, fatigue, cough, or diarrhea, would likely first visit an IP. During disease outbreaks there is concern about how well health care providers are informed about the disease and its management, particularly when knowledge about the disease is rapidly changing. During previous outbreaks, such as Severe Acute Respiratory Syndrome (SARS) and Middle East Respiratory Syndrome (MERS), collecting real-time information on provider knowledge and understanding of an emerging disease case management has supported response efforts by improving triage procedures and reducing infections in health care settings [6]–[8]. Like SARS and MERS, COVID-19 is a coronavirus disease, spread by airborne transmission from close personal contact. Despite the large presence of IPs in the primary care workforce of many LMIC countries, little is known about their knowledge and practices related to COVID-19.

IPs have no formal medical training from a recognized institution, though they commonly have some form of informal training [3]. In several countries, IPs are a substantial presence in the health workforce - they constitute around 88% of all health care providers in Bangladesh, and 77% in Uganda [3]. In India most curative health services are provided by the private sector, and paid for out-of-pocket due to limited financial protection coverage, despite the fact that free care is available at government clinics [9]. India's pluralistic health system has a variety of formal and informal primary care providers – IPs, qualified AYUSH[1] physicians, and allopathic doctors. IPs comprise between 24% to 43% of the health workforce in India, and their share of the health workforce varies greatly between and within states [10], [11]. IPs comprise a large share of the rural primary care health workforce - around 70% in rural compared to 31% of urban clinical care providers [11]. Smaller scale state studies also indicate a large presence of IPs – for example one study found that in a district in the state of Karnataka 74% of the clinical care providers were IPs, while in a district in Uttarakhand, their share was 79% [4], [11]. In general, IPs are trusted community members who practice within villages and charge fees-for-services which are paid for out-of-pocket. People seek care from IPs for a number of reasons, including trust in the care IPs provide, proximity, and lower cost relative to formally trained private providers [12].

Indian IPs are a heterogenous group of medical providers who can practice allopathic or Indian systems of medicines, or a mix of these [4]. IPs typically treat common illnesses like fever, diarrhea, and respiratory conditions and play an important role in referring cases to higher-level health facilities [4]. The few studies on IPs in India report a range of clinical experience – including certificate courses in allopathic and Indian systems of medicine, or apprenticeships with qualified doctors [4]. Studies on IP treatment practices find that they produce poor quality care, though they might be knowledgeable about treatment protocols

[1] AYUSH are trained practitioners of Indian systems of medicine and homeopathy i.e. Ayurveda, Yoga, Unani, Siddha and Homeopathy.

For peer review only - http://bmjopen.bmj.com/site/about/guidelines.xhtml

[3]. Interestingly, studies that have compared IPs with qualified primary care doctors find only small differences between them in protocol adherence, and no differences in the likelihood of giving a correct diagnosis or treatment [13].

Over the past year, several studies have assessed healthcare provider knowledge and perceptions of COVID-19. These studies have focused on qualified providers and report good knowledge of COVID-19, though there are important gaps to be addressed as best practices evolve over time [14]. Studies among health providers in low- and middle-income settings have echoed the broader literature by reporting good knowledge of COVID-19 symptoms and prevention, but gaps in knowledge of case management protocols [15], [16]. In India, studies on the knowledge and practices of health care professionals such as doctors, medical residents, medical students, and other formally trained health workers reported high levels of knowledge of COVID-19 symptoms, and preventive measures, but suggested lower levels of proficiency in terms of case management [17]–[19]. To the best of our knowledge, no study has attempted to document knowledge and practices related to COVID-19 among IPs. In general, previous studies during SARS and MERS outbreaks reported that health care providers had good understanding of disease symptoms and prevention but did poorly on protocols for case management [20]–[23].

In this study we aim to understand the knowledge and practices of formal and informal primary care providers – IPs, AYUSH physicians, and allopathic doctors - related to COVID-19. Our study is based on a telephonic survey of primary care providers in the state of Bihar in eastern India. Understanding the knowledge and practices of IPs and other primary care providers in the context of the COVID-19 pandemic has important implications for the health care that communities receive, and more importantly, for the government's pandemic response.

Methods

This paper is based on a cross sectional survey of primary health care providers in rural Bihar conducted via telephonic interviews. The survey was conducted from 1st to 15th July 2020, a period of rapid increase in the COVID-19 cases in the state.

Setting: With a population of over 100 million and a GDP per capita of US $640 (compared with the national GDP per capita of US $2,099) Bihar is among India's resource poor states. It's residents are spread across 38 districts and some 45,000 villages, 88% of which are considered to be located in rural areas [24]. Although the state has made important gains in population health over the last few decades, it remains among the poorer performing states of India. Bihar's health system is under resourced, including its human resources for health workforce, which is operating at 1.5 health workers per 10,000 population, well under WHO's recommended 22.8 workers per 10,000 population [11]. Bihar's pluralistic health system is characterized by a large presence of IPs, particularly in rural areas. This shortage of health workers and dependence on informal providers has hampered Bihar's ability to deal with the COVID-19 outbreak [25]. At the time of this study, the first half of July 2020, Bihar was experiencing a rapid increase in the number of recorded COVID-19 cases. Confirmed cases increased from around 400 cases per day at the beginning of July to about 1,300 cases per day by mid-July. Daily new cases continued to steadily increase to a peak of 3,900 new cases in mid-August. From the beginning of July to end of August, India experienced more

For peer review only - http://bmjopen.bmj.com/site/about/guidelines.xhtml

than a three-fold increase in cases, from approximately 19,000 to 70,000 new cases per day. This came even after India instituted one of the strictest national lockdowns in the world which lasted from mid-March until the end of May. Under the lockdown, people were restricted from leaving their homes and all transport services, educational institutions, and hospitality services were suspended- violators were punishable by up to a year in jail. The lockdown severely affected the national economy and forced thousands of migrant workers to return to Bihar from cities across India. The spread of COVID-19 in rural Bihar has in part been attributed to the return of these migrant workers [26].

Study sample: The primary care providers in this study were identified from a parent household survey conducted in rural Bihar between November 2019 and March 2020. This parent survey's objective was to understand primary care seeking patterns in rural Bihar. In this survey, 70 blocks (of 534 total blocks) across Bihar's nine divisions were selected using stratified systematic random sampling. Within each block, five villages were selected using probability proportional to size sampling. In each village, a probability sample of 30 households was selected using segmented random sampling. The household survey covered 70 blocks and 343 villages across 37 districts in Bihar; a total of 8,356 households, and 39,477 individuals were sampled. Of the individuals sampled, 15,811 (40%) reported being ill in the past month, and 10,617 (71%) of them sought care outside their home.

Respondents who sought care outside home were asked to report details of the providers they visited. We collected phone numbers and geolocations of these providers (if they were within 5 kilometers of the village) with the idea of surveying these providers at a later date to assess aspects of quality of care. Given the outbreak of coronavirus in March 2020 in India, the provider survey had to be suspended. However, we felt that we could contribute to the state's COVID-19 response by contacting these providers via telephone to understand their experiences during the outbreak. Any provider identified through the parent study was eligible for inclusion in the telephone survey.

A total of 9,497 provider contacts were recorded in the household survey. Of these, we had complete contact information for 6,717 providers. After cleaning to remove drug shops (1,603), community health workers (35), and duplicate providers (3,941), we obtained telephone numbers of 1,138 private providers across 256 villages. We made three attempts to contact each respondent. Of the total of 1,138 providers contacted, we were able to successfully interview 522 private providers across 224 villages, achieving a 46% response rate. The key reasons for non-response included invalid telephone number (28%), respondent not interested in participating (19%), phone switched off (15%), and no response to calls (12%). At each block level Primary Health Center (PHC) in the 70 blocks covered by the household survey, the PHC Medical Officer was contacted and included in our study. Of the 70 PHCs contacted, we were able to conduct telephone interviews with from 25 PHC Medical Officers, which translates to a 36% response rate.

Data collection: Providers were interviewed using a structured questionnaire with choice-based answers to gather information on (i) change in patient care seeking, (ii) source of COVID-19 information, (iii) knowledge on COVID-19 spread, symptoms, and methods for prevention, and (iv) clinical management of COVID-19. Where provider answers were ambiguous, enumerators were trained to probe the respondent to reach a clear answer, after which enumerators made a judgement on the most appropriate answer choice among the

For peer review only - http://bmjopen.bmj.com/site/about/guidelines.xhtml

available selections. Given the challenge of keeping the respondent engaged in a telephonic survey, every attempt was made to keep the tool short and precise. Average time taken to complete an interview was around 20 minutes. The phone surveys were carried out by Oxford Policy Management, Delhi. All enumerators possessed a nursing degree, had prior experience in conducting quantitative interviews, and were trained to conduct telephonic surveys using computer-assisted telephonic interviewing (CATI) software integrated with CS Pro [27]. The CATI software displays the questionnaire on the screen of a tablet and the interviewer records the answers on the tablet during the interview. It also records the calls between the interviewer and provider enabling spot checks at a later date. Using CATI minimizes information bias as the skip logic is already embedded in the questionnaire and data is automatically recorded in a data management platform, removing the need for double data entry.

Data Analysis: A random subset of the calls recorded were checked by a data manager to identify any errors and mismatches with the data entered in CS Pro. Variables of interest were mostly categorical and for most questions, respondents could select more than one response option. Respondents were classified according to their self-reported medical training. A provider was classified as an IP if they served as providers in a private facility and reported their training as any of the following - Registered Medical Practitioner (RMP), no formal qualification, diploma in modern and holistic medicine, nurse, pharmacist, community health worker, and a range of other non-degree qualifications. Providers who reported being trained in Indian systems of medicine were classified as AYUSH doctors. Providers who said they had a Bachelor of Medicine, Bachelor of Surgery (MBBS) or higher degree were classified as MBBS doctors. We conducted exploratory data analysis on the variables of interest to identify response patterns by provider types. Univariate and bivariate statistics were computed for variables of interest using two sample t-tests or chi-square tests of significance across provider types where necessary. Graphical analysis of the data was carried out. Missing data (responses from 2 IPs) was not included in the analysis. Each provider was treated as an independent observation. Statistical analysis was conducted using Stata 14 [28].

Ethical approval: Ethical approval was granted by the Sigma Institutional Review Board in India (Reference number: 1007/IRB/20-21) as well as by the Johns Hopkins University Institutional Review Board. The purpose of the study was explained and oral informed consent was obtained from each respondent. If the respondent agreed to participate, then a signed copy of the consent form was sent to the respondent via Short Message Service (SMS) text or WhatsApp.

Patient and public involvement: Questions for the household survey were pre-tested on the general public, including patients, and were appropriately modified. Reports of where sick household members sought treatment enabled identification and recruitment of primary care providers for this study. Further, the questionnaire was tailored so that it would not place an excessive burden on providers.

Results

The 547 providers included in our sample were from 224 villages across 34 of Bihar's 38 districts. Our sample included 452 (83%) IPs, 57 (10%) AYUSH providers, and 38 (7%) MBBS doctors. Of these, 522 (95%) were private and 25 (5%) were public providers (Table

For peer review only - http://bmjopen.bmj.com/site/about/guidelines.xhtml

1). All public providers were located either at a primary health care facility or a community health center.

[Table 1 here]

Providers were asked if they were still seeing patients despite the nation-wide lockdown. Among IPs, 73% reported seeing patients in person, 5% reported consulting patients over the phone, and 22% reported not seeing patients in the last week. AYUSH doctors reported slightly higher rates of physical patient interactions, with 79% seeing patients, 4% consulting patients over the phone, and 18% not seeing any patients in the last week. Among MBBS doctors, 86% were seeing patients in person, 5% consulted patients over the phone, and 8% did not see patients in the last week. A higher percent of public MBBS doctors saw patients in the last week than private MBBS providers (91% vs 80%), and while a similar percent of MBBS providers did not see any patients in the last week (9% of public vs 7% of private), 13% of private MBBS doctors consulted patients over the phone, while no publicly employed MBBS doctors employed this technique. We asked providers who were still seeing patients if there was any change in the volume of patient visits the week before the survey as compared to what they usually experience (Figure 1). The majority of providers (72%), irrespective of qualification or public or private sector, reported a fall in patient visits. However, nearly one fifth of IPs reported no change in patient volume over the prior week as compared to normal business. Public sector providers most frequently reported an increase in patient visits in the previous week.

[Figure 1 here]

A small (7%) proportion of private providers, including IPs, reported receiving training related to COVID-19 from either government or civil society sources, compared to 72% of public providers. Knowing where providers get their information on COVID-19 is important to plan future health communications activities (Table 2). Television was the most common source of information for all provider types except public MBBS doctors, for whom it was the second most common information source. For IPs, AYUSH providers, and private MBBS providers, newspapers were the second most common source of information. Nearly all (95%) of the public MBBS providers reported receiving information from government sources, compared to 29% of private MBBS providers, 34% of IPs, and 35% of AYUSH doctors. Interestingly, mobile phones and the radio did not feature as important information sources across provider types.

[Table 2 here]

We asked providers to name common COVID-19 symptoms (Table 2). Overall, there was no significant difference in knowledge across provider types. The vast majority of providers in each group were able to identify symptoms such as fever, cough, and breathing problems. In contrast, diarrhea was far less frequently identified as a symptom of COVID-19 across provider types. Interestingly, nearly a third of public MBBS providers identified loss of taste or smell as a COVID-19 symptom – a far greater percent than any other provider type.

Common public health measures for preventing COVID-19 infection, such as using a facemask, washing hands and distancing from other people were widely known across

For peer review only - http://bmjopen.bmj.com/site/about/guidelines.xhtml

provider types (Table 2). Others such as staying indoors or avoid touching one's face were less frequently reported.

Of interest is to know how primary care providers in Bihar would manage a suspected case of COVID-19. We asked providers *"In the past week, if a patient came to you with fever, cough, and breathing difficulty, what would you tell them to do?"* According to the WHO ("Clinical Management of COVID-19: Interim guidance, May 27 2020") and Government of India (Clinical Management Protocol: COVID-19) guidelines, such a person would be a COVID-19 suspect having mild to moderate symptoms [29], [30]. We classified provider responses in terms of the WHO-recommended actions for providers when presented with a suspected case of COVID-19 having mild to moderate symptoms (Figure 2). The Government of India guidelines had substantial overlap with these WHO-recommended actions. Overall, there was no significant difference between IPs and other provider types in following recommended actions, except for prescribing fever medication. A little more than half the primary care providers said they would require the patient to wear a mask during consultation. The majority said they would tell the patient to take a COVID-19 test. Testing advice was highest for IPs (69%), followed by AYUSH (63%), and MBBS doctors (63%). Prescribing fever medication to the patient varied significantly across provider types. Around 79% of MBBS doctors said they would prescribe fever medication, compared to around half the IPs and 39% of AYUSH providers. Advice about monitoring for complications was low across provider types – 44% of AYUSH, 41% of IPs, and 31% of MBBS doctors said they would ask the patient to check if the symptoms became worse after a few days. Assessing patients for risk factors of severe complications, such as existing health conditions of heart disease or diabetes, was reported by less than half the providers in any group – 44% AYUSH, 38% IPs and 31% MBBS doctors. There was no significant difference across provider types in assessing patients for risk factors associated with complications. Advising patients to isolate at home was recommended by around 60% of IPs and MBBS doctors, compared to 50% for AYUSH providers. Overall, only 16% of IPs, 15% of MBBS doctors, and 12% of AYUSH practitioners reported all these recommended actions. On average, providers complied with 60% of the six recommended actions.

[Figure 2 here]

We asked providers if they would refer a patient who came to them in the past week with symptoms of fever, cough, and breathing difficulty to a higher level health facility (Figure 3). Nearly half the providers in each group said they would not refer such patients. Across provider types, among those who said they would refer, government clinics or hospital were the preferred places for referral (66% of referrals were to government clinics or hospitals). There was no significant difference across groups in referral patterns.

[Figure 3 here]

Discussion

Responding to pandemics is particularly challenging in pluralistic health systems. When diverse systems of medicine are practiced and there is a mix of informal and formal health workers, it is a challenge to achieve uniform standards in providers' understanding of the pandemic, ways to prevent infection, and patient case management. This challenge is

For peer review only - http://bmjopen.bmj.com/site/about/guidelines.xhtml

exacerbated by the ambiguous space that IPs occupy in India's health policy. One view, which reflects prevailing policy attitudes, is that IPs pose a danger to patients, and represent a problem that needs to be addressed. The alternative view is that they fill a vacuum in primary care service provision, and since they are already embedded within communities, it is pragmatic to engage with them. Findings from our study reflect both perspectives. Rural primary care providers as a whole were relatively well informed about the basics of COVID-19 symptoms and preventive measures, but performed poorly in terms of following recommended case management actions. In most cases, IPs performed similarly to MBBS or AYUSH doctors, but their low level of compliance could still endanger patients. On the other hand, over half of IPs recommended referring a suspect case to a government or other health clinic, so IPs could provide an important link to more sophisticated care. As the COVID-19 pandemic spreads across rural India, IPs will likely to the first contact providers for many patients; as such, there is much to be gained if appropriate actions are taken by them in patient encounters. Further, because IPs are embedded in rural communities, they can play an important role in contact tracing, and in public health messaging.

One of the significant, though not unexpected, findings from this study was the lack of contact (or training) that IPs and other private primary care providers had with government or civil society agencies. One consequence of this is that COVID-19 knowledge for most IPs and other primary care providers was primarily coming from TV and newspaper sources. In contrast, most medical doctors in the government system reported receiving information on COVID-19 directly from government sources. Despite the lack of government engagement, IPs and other primary care providers were remarkably well informed of certain COVID-19 symptoms and preventive measures. This echoes findings from previous studies on the COVID-19 knowledge of qualified health professionals [15], [16]. Importantly, this finding highlights the importance and responsibility of popular media sources in providing public health messaging to rural clinical providers. However, reliance on popular media alone may not be adequate. For example, providers had low awareness about symptoms, especially diarrhea and the lack of taste or smell, which were described in the medical community as early as May 2020 and are now recognized as important COVID-19 symptoms [31]. Further, the importance of referral to clinics and testing sites could be further emphasized to better understand the local impact of the pandemic. While the frequency of referral between IPs and formal providers largely relies on established relationships and incentive structures, referral for COVID-19 testing could be an opportunity to strengthen linkages between the informal and formal sector [4]. As such, there is a role for government in providing health information to primary care providers, particularly in the context of a pandemic.

Engaging private practitioners embedded in local communities has been an important strategy for controlling the COVID-19 pandemic. In the urban slum of Dharavi in Mumbai, one of the largest slum areas of the world, local government agencies have effectively controlled the COVID-19 outbreak using a range of measures, including using local health practitioners to engage their communities with public health messaging, screening, contact tracing, and providing clinical services [32]. Community trust in local health care providers considerably aided government efforts in the pandemic response. In Bihar, and elsewhere in India, there have been earlier efforts by government and civil society organizations to engage with IPs and other private sector providers to improve quality of care [33]. Such actions have not yet been taken for the COVID-19 response [34]. Clearly, providing information to IPs (and other private providers) on COVID-19, its prevention, and where testing centers are located can

For peer review only - http://bmjopen.bmj.com/site/about/guidelines.xhtml

help improve community knowledge on preventive practices and management of suspected cases. Since IPs are embedded in local communities, they are an invaluable resource for contact tracing and sharing public health messaging on COVID-19 to rural communities.

Our study finds that rural primary care providers had generally poor (stated) compliance with recommended case management practices for COVID-19. Their stated actions when presented with a suspected COVID-19 case, such as advising the patient to wear a mask, getting a COVID-19 test, prescribing fever medication, monitoring the patient for complications, asking patients about risk factors, and advising isolation were generally not practiced by IPs and more importantly, not by formal providers, as the onus is on the formally trained. In fact, only a small minority of primary care providers stated that they would do all these recommended practices. This points to the poor COVID-19 case management practices among formally trained providers and IPs alike. AYUSH and MBBS doctors, from both public and private facilities, did not perform significantly better than IPs. The observation that IPs (or AYUSH providers) don't differ significantly from MBBS doctors in their stated practices confirms similarities in quality of care that have been reported in other studies [35]. Findings from our study are likely generalizable to other rural contexts in resource limited states in India.

There are two notable limitations to our study. First, the high level of non-responses (55%), while common in telephone surveys, raises concerns about selection bias due to providers with certain characteristics not participating. There is some evidence for this – for example, among MBBS doctors there appears to be a higher non-response among public (75%) compared to private sector (46%) providers. If this non-response is related to the competency of respondents i.e. more competent doctors did not participate, then our estimates of compliance with quality actions would be biased in the negative direction. However, studies report that in physician surveys, the extent of bias due to non-response is likely minimal because physicians are quite homogenous as a group in terms of knowledge and training, and variations that are present between them are unlikely to be associated with their likelihood of responding [36]. A second concern relates to the assessment of COVID-19 case management; which is based on what respondents said they would do. For one, reported actions could differ from what providers actually do in practice. Studies that have examined differences in knowledge and practice have found significant gaps, particularly among more knowledgeable providers [37], [38]. While it is difficult to judge how well provider-stated intentions are reflected in practice, these stated actions can be viewed as an upper limit of what they might do in practice. Further, in the context of a telephone interview, it is possible that there was underreporting of some key actions providers might take in practice because of trust issues. For example, it is somewhat surprising that so few IPs and AYUSH providers, who cannot officially prescribe allopathic medications, said they would prescribe fever medication (which is widely available) to someone with COVID-19 symptoms. While these providers could have answered that they prescribed other medicines such as cough medicines or antibiotics, about one fifth of all IPs and AYUSH providers answered that they would not prescribe any medicines.

In pluralistic health systems where IPs and other private providers comprise a large share of primary care providers, embracing the entire health workforce in the government's COVID-19 response offers several advantages. Because IPs are likely to be the first contact primary care provider for the majority of symptomatic individuals, their management of suspect cases

For peer review only - http://bmjopen.bmj.com/site/about/guidelines.xhtml

becomes vital to state and national efforts to control the outbreak. Moreover, because they are embedded within communities, IPs can assist in contact tracing, and public health messaging. As such, IPs can be an important partner in the government's COVID-19 response.

For peer review only

Author contribution: KDR, JK, MAP and PN were responsible for conceiving and designing the study. JK and NK were responsible for organizing data collection. KDR, JK, and MAP contributed to, data analysis. All authors contributed to writing and editing the manuscript.

Data sharing: Please contact Krishna D. Rao (kdrao@jhu.edu) for data related to this study.

Conflict of interest: None declared for KDR, JK, MKP, and NK. PN is employee of the funding agency.

Funding: Bill and Melinda Gates Foundation, Delhi. Grant # OPP119434. One of the authors (PN) is employed by the funder.

For peer review only - http://bmjopen.bmj.com/site/about/guidelines.xhtml

References

[1] B. Meessen *et al.*, "Composition of pluralistic health systems: how much can we learn from household surveys? An exploration in Cambodia," *Health Policy Plan.*, vol. 26, no. suppl_1, pp. i30–i44, Jul. 2011.

[2] G. Bloom and H. Standing, "Pluralism and marketisation in the health sector: meeting health needs in contexts of social change in low and middle-income countries," 2001.

[3] M. Sudhinaraset, M. Ingram, H. K. Lofthouse, and D. Montagu, "What Is the Role of Informal Healthcare Providers in Developing Countries? A Systematic Review," *PLoS One*, vol. 8, no. 2, Feb. 2013.

[4] M. Gautham, K. M. Shyamprasad, R. Singh, A. Zachariah, R. Singh, and G. Bloom, "Informal rural healthcare providers in North and South India," *Health Policy Plan.*, vol. 29, no. SUPPL. 1, p. i20, 2014.

[5] R. Priya, R. Singh, and S. Das, "Health Implications of Diverse Visions of Urban Spaces: Bridging the Formal-Informal Divide," *Front. Public Heal.*, vol. 7, p. 239, Sep. 2019.

[6] J. F. Deng *et al.*, "Severe acute respiratory syndrome (SARS): Knowledge, attitudes, practices and sources of information among physicians answering a SARS fever hotline service," *Public Health*, vol. 120, no. 1, pp. 15–19, 2006.

[7] A. J. Alsahafi and A. C. Cheng, "Knowledge, Attitudes and Behaviours of Healthcare Workers in the Kingdom of Saudi Arabia to MERS Coronavirus and Other Emerging Infectious Diseases," *Int. J. Environ. Res. Public Health*, vol. 13, no. 12, p. 1214, Dec. 2016.

[8] S. Al-Amri, R. Bharti, S. A. Alsaleem, H. M. Al-Musa, S. Chaudhary, and A. A. Al-Shaikh, "Knowledge and practices of primary health care physicians regarding updated guidelines of MERS-CoV infection in Abha city," *J. Fam. Med. Prim. care*, vol. 8, no. 2, pp. 455–461, Feb. 2019.

[9] Government of India, Ministry of Statistics & Programme Implementation, and National Statistical Office, "Health In India: National Sample Survey 75th Round," 2018.

[10] A. Karan, H. Negandhi, R. Nair, A. Sharma, R. Tiwari, and S. Zodpey, "Size, composition and distribution of human resource for health in India: New estimates using National Sample Survey and Registry data," *BMJ Open*, vol. 9, no. 4, p. e025979, Apr. 2019.

[11] K. D. Rao, R. Shahrawat, and A. Bhatnagar, "Composition and distribution of the health workforce in India: estimates based on data from the National Sample Survey," *WHO South-East Asia J. public Heal.*, vol. 5, no. 2, pp. 133–140, Sep. 2016.

[12] G. Anand, D. Chhajed, S. Shah, S. Atkins, and V. Diwan, "Do qualifications matter? A qualitative study of how villagers decide their health care providers in a developing economy," *PLoS One*, vol. 14, no. 8, p. e0220316, Aug. 2019.

[13] J. Das, A. Holla, V. Das, M. Mohanan, D. Tabak, and B. Chan, "In urban and rural India, a standardized patient study showed low levels of provider training and huge quality gaps," *Health Aff.*, vol. 31, no. 12, pp. 2774–2784, 2012.

[14] I. M. Puspitasari, L. Yusuf, R. K. Sinuraya, R. Abdulah, and H. Koyama, "Knowledge, Attitude, and Practice During the COVID-19 Pandemic: A Review," *J. Multidiscip. Healthc.*, vol. Volume 13, pp. 727–733, Jul. 2020.

[15] G. Huynh, T. Nguyen, V. Tran, K. Vo, V. Vo, and L. Pham, "Knowledge and attitude toward COVID-19 among healthcare workers at District 2 Hospital, Ho Chi Minh

For peer review only - http://bmjopen.bmj.com/site/about/guidelines.xhtml

City," *Asian Pac. J. Trop. Med.*, vol. 13, no. 6, pp. 260–265, Jun. 2020.

[16] R. Olum, G. Chekwech, G. Wekha, D. R. Nassozi, and F. Bongomin, "Coronavirus Disease-2019: Knowledge, Attitude, and Practices of Health Care Workers at Makerere University Teaching Hospitals, Uganda," *Front. Public Heal.*, vol. 8, Apr. 2020.

[17] S. Mehrotra, P. Jambunathan, M. Jindal, A. Gupta, and K. Kapoor, "A cross-sectional survey to assess the knowledge regarding coronavirus disease (COVID-19) among health care professionals," *Med. J. Armed Forces India*, 2020.

[18] J. Kochuparambil, A. Issac, S. George, and N. Panicker, "Knowledge pertaining to COVID19 among medical population of Indian state of Kerala: An online cross-sectional survey," *J. Patient Saf. Infect. Control*, vol. 8, no. 1, p. 17, 2020.

[19] R. P. Kotian, D. Faujdar, S. P. Kotian, and B. D'souza, "Knowledge and understanding among medical imaging professionals in India during the rapid rise of the covid-19 pandemic," *Health Technol. (Berl).*, p. 1, May 2020.

[20] A. Asaad, R. El-Sokkary, M. Alzamanan, and M. El-Shafei, "Knowledge and attitudes towards middle east respiratory sydrome-coronavirus (MERS-CoV) among health care workers in South-Western Saudi Arabia," *East. Mediterr. Heal. J.*, vol. 26, no. 4, pp. 435–442, Apr. 2020.

[21] M. U. Khan, S. Shah, A. Ahmad, and O. Fatokun, "Knowledge and attitude of healthcare workers about middle east respiratory syndrome in multispecialty hospitals of Qassim, Saudi Arabia," *BMC Public Health*, vol. 14, no. 1, 2014.

[22] A. Bener and A. Al-Khal, "Knowledge, attitude and practice towards SARS," *J. R. Soc. Promot. Health*, vol. 124, no. 4, pp. 167–170, Jul. 2004.

[23] A. D. Tice, M. Kishimoto, C. H. Dinh, G. Tak-Kin Lam, and M. Marineau, "Knowledge of Severe Acute Respiratory Syndrome among Community Physicians, Nurses, and Emergency Medical Responders," *Prehosp. Disaster Med.*, vol. 21, no. 3, pp. 183–189, Jun. 2006.

[24] Office of the Registrar General & Census Commissioner, "Census of India," 2011. [Online]. Available: http://www.censusindia.gov.in/2011census/hh-series/hh01.html. [Accessed: 03-Dec-2019].

[25] A. Kumar and M. Kumar, "Covid-19 and the public health system in Bihar," *Econ. Polit. Wkly.*, 2020.

[26] K. Mehrotra, "Over a month, Covid hits more districts, most in Bihar and UP," *The Indian Express*, New Delhi, May-2020.

[27] United States Census Bureau, "CS Pro," 2020. [Online]. Available: https://www.census.gov/data/software/cspro.html.

[28] StataCorp, "Stata Statistical Software Release 14," College Station, TX., 2014.

[29] Ministry of Health and Family Welfare, "National Clinical Management Protocol: COVID-19 ," *Version 5*, 03-Jul-2020. [Online]. Available: https://www.mohfw.gov.in/pdf/UpdatedClinicalManagementProtocolforCOVID19dated03072020.pdf. [Accessed: 20-Oct-2020].

[30] World Health Organization, "Clinical management of COVID-19," 27-May-2020. [Online]. Available: https://www.who.int/publications/i/item/clinical-management-of-covid-19. [Accessed: 20-Oct-2020].

[31] C. Menni *et al.*, "Real-time tracking of self-reported symptoms to predict potential COVID-19," *Nat. Med.*, vol. 26, no. 7, pp. 1037–1040, 2020.

[32] M. Golechha, "COVID-19 Containment in Asia's Largest Urban Slum Dharavi-Mumbai, India: Lessons for Policymakers Globally," *J. Urban Heal.*, vol. 97, no. 6,

For peer review only

pp. 796–801, 2020.

[33] M. Mohanan, K. S. Babiarz, J. D. Goldhaber-Fiebert, G. Miller, and M. Vera-Hernández, "Effect of a large-scale social franchising and telemedicine program on childhood diarrhea and pneumonia outcomes in India," *Health Aff.*, 2016.

[34] M. Gautham, G. Bloom, P. Balasubramaniam, C. Goodman, B. C. Forsberg, and A. Kumar, "Mobilising informal healthcare providers in India may help its response to covid-19," *The BMJ Opinion*, 2020. [Online]. Available: https://blogs.bmj.com/bmj/2020/06/24/mobilising-informal-healthcare-providers-in-india-may-help-its-response-to-covid-19/.

[35] J. Das, B. Daniels, M. Ashok, E.-Y. Shim, and K. Muralidharan, "Two Indias: The structure of primary health care markets in rural Indian villages with implications for policy," *Soc. Sci. Med.*, p. 112799, Jun. 2020.

[36] S. E. Kellerman and J. Herold, "Physician response to surveys: A review of the literature," *American Journal of Preventive Medicine*, vol. 20, no. 1. Elsevier Inc., pp. 61–67, 2001.

[37] K. L. Leonard and M. C. Masatu, "The use of direct clinician observation and vignettes for health services quality evaluation in developing countries," vol. 61, pp. 1944–1951, 2005.

[38] M. Mohanan, M. Vera-hernández, V. Das, S. Giardili, and A. Seth, "The Know-Do Gap in Quality of Health Care for Childhood Diarrhea and Pneumonia in Rural India," vol. 27708, no. 4, pp. 349–357, 2015.

For peer review only

Table 1: Sample characteristics

	Private Providers	Public Providers	Total providers
MBBS	15	23	38
AYUSH	55	2	57
Informal Provider	452	0	452
Total	522	25	547

Table 2: Source of information and knowledge of COVID-19

	IP (%)	Private MBBS (%)	Public MBBS (%)	AYUSH (%)	Total (%)
N(providers)	452	15	23	57	547
**Sources of information**					
TV	76	80	52	74	75
Newspaper	53	53	35	40	51
Government	34	27	91	35	36
Friends	24	20	4	23	23
Mobile phone	12	7	4	7	11
Radio	9	7	9	14	10
**Knowledge of COVID-19 symptoms**					
Fever	90	100	96	89	90
Cough	83	93	87	86	84
Breathing problem	71	67	83	79	72
Body ache	24	13	35	25	24
Sore throat	23	7	17	26	23
Fatigue	15	13	9	12	15
Diarrhea	6	13	13	9	7
Loss or taste/smell	8	13	30	5	9
**Knowledge of COVID-19 prevention**					
Use facemask	83	100	83	79	83
Washing hands	80	87	91	74	80
Social distance	76	67	87	79	77
Stay at home	15	33	17	18	16
Avoid touching face	11	7	17	12	11

Note: Respondents can select multiple responses. There were two missing values (both IPs) for sources of information, or knowledge of COVID-19 symptoms, or knowledge of COVID-19 prevention.

For peer review only - http://bmjopen.bmj.com/site/about/guidelines.xhtml

Figure 1: Percentage of providers reporting change in patient visits during COVID-19 outbreak

Figure 2: Provider stated compliance with WHO recommended actions for COVID-19 suspects

Figure 3: Referral patterns for COVID-19 suspect cases

For peer review only

For peer review only - http://bmjopen.bmj.com/site/about/guidelines.xhtml

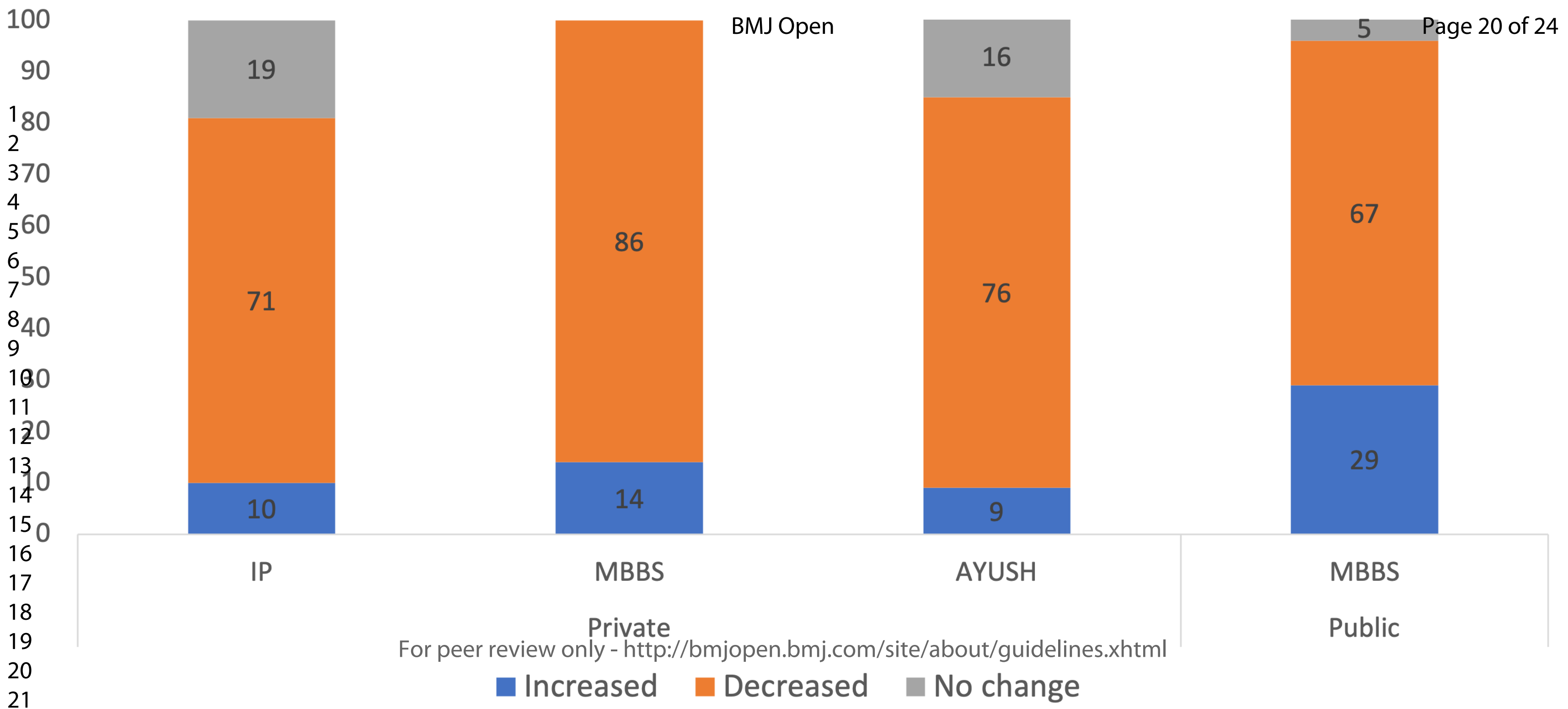
100
90
80
70
60
50
40
30
20
10
0
19
71
10
86
14
16
76
9
5
67
29
IP
MBBS
AYUSH
MBBS
Private
Public
Increased
Decreased
No change

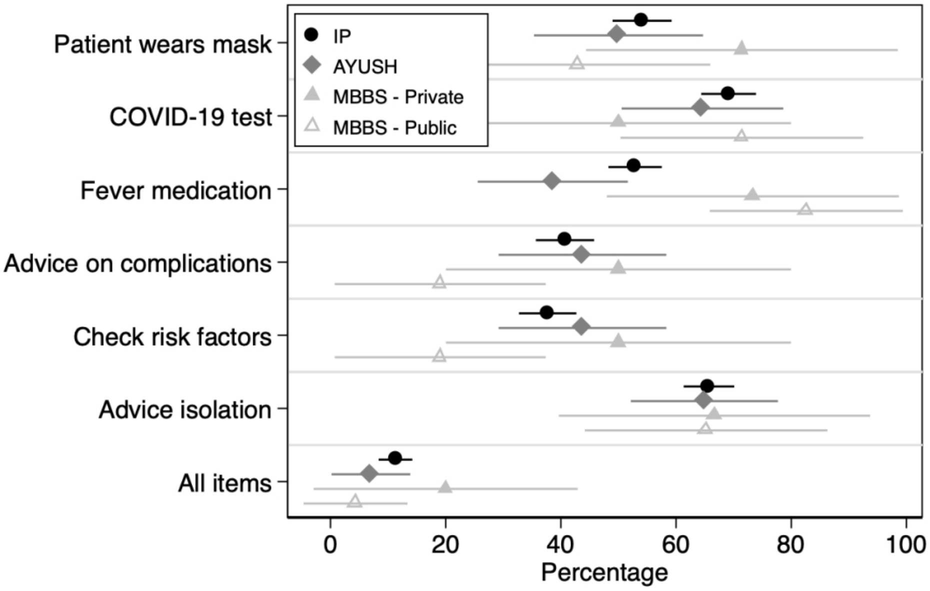

Note: (1) IP is Informal provider, MBBS is a provider with an MBBS degree, AYUSH is provider with a degree. in Indian systems of medicine or homeopathy.
(2) Figures represent point estiamtes and 95% confidence intervals.
(3) Recommendations from Clinical management of COVID-19, Interim guidance, WHO, May 27, 2020.

Figure 2: Provider stated compliance with WHO recommended actions for COVID-19 suspects

239x174mm (144 x 144 DPI)

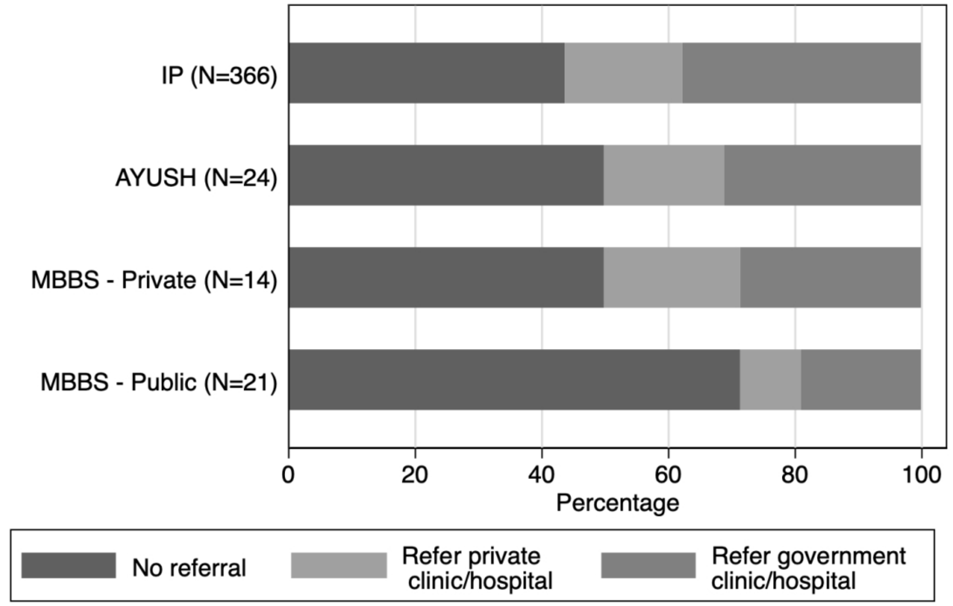

Note: Above resonses are to the question: In the past week if a patient came to you with symptoms of fever, cough, and breathing difficulty would you refer this patient to another doctor?
Question was not asked to providers who said they did not see patients in the last week

Figure 3: Referral patterns for COVID-19 suspect cases

261x190mm (144 x 144 DPI)

[1]STROBE Statement—Checklist of items that should be included in reports of ***cross-sectional studies***

	Item No	Recommendation	Action
**Title and abstract**	1	(*a*) Indicate the study's design with a commonly used term in the title or the abstract	Page 1/line 4
		(*b*) Provide in the abstract an informative and balanced summary of what was done and what was found	Page 2/line 8-47
**Introduction**			
Background/rationale	2	Explain the scientific background and rationale for the investigation being reported	Page 4/lines 3-39 Page 5/ lines 6-19
Objectives	3	State specific objectives, including any prespecified hypotheses	Page 5/lines 21-27
**Methods**			
Study design	4	Present key elements of study design early in the paper	Page 5/lines 33-35
Setting	5	Describe the setting, locations, and relevant dates, including periods of recruitment, exposure, follow-up, and data collection	Page 5 line 37 – page 6 line 11
Participants	6	(*a*) Give the eligibility criteria, and the sources and methods of selection of participants	Page 6/line 13-44
Variables	7	Clearly define all outcomes, exposures, predictors, potential confounders, and effect modifiers. Give diagnostic criteria, if applicable	Page 6 line 46 – Page 7 line 14
Data sources/ measurement	8*	For each variable of interest, give sources of data and details of methods of assessment (measurement). Describe comparability of assessment methods if there is more than one group	Page 6 line 46 – Page 7 line 14
Bias	9	Describe any efforts to address potential sources of bias	Page 7/line 12-14 Page 11/line 24-34
Study size	10	Explain how the study size was arrived at	Page 6/line 13-44
Quantitative variables	11	Explain how quantitative variables were handled in the analyses. If applicable, describe which groupings were chosen and why	Page 7/lines 16-31
Statistical methods	12	(*a*) Describe all statistical methods, including those used to control for confounding	Page 7/lines 16-31
		(*b*) Describe any methods used to examine subgroups and interactions	NA
		(*c*) Explain how missing data were addressed	Page 7/lines 29-31
		(*d*) If applicable, describe analytical methods taking account of sampling strategy	NA
		(*e*) Describe any sensitivity analyses	None reported

Results

For peer review only - http://bmjopen.bmj.com/site/about/guidelines.xhtml

For peer review only

Participants	13*	(a) Report numbers of individuals at each stage of study—eg numbers potentially eligible, examined for eligibility, confirmed eligible, included in the study, completing follow-up, and analysed	Page 8/ lines 1-5
		(b) Give reasons for non-participation at each stage	Page 6/lines 38-40
		(c) Consider use of a flow diagram	NA
Descriptive data	14*	(a) Give characteristics of study participants (eg demographic, clinical, social) and information on exposures and potential confounders	Page 8/ lines 1-5
		(b) Indicate number of participants with missing data for each variable of interest	Page 7/line 29
Outcome data	15*	Report numbers of outcome events or summary measures	Page 8 line 1 – Page 9 line 42
Main results	16	(*a*) Give unadjusted estimates and, if applicable, confounder-adjusted estimates and their precision (eg, 95% confidence interval). Make clear which confounders were adjusted for and why they were included	Page 8 line 1 – Page 9 line 42
		(*b*) Report category boundaries when continuous variables were categorized	Page 7 / lines 19-26
		(*c*) If relevant, consider translating estimates of relative risk into absolute risk for a meaningful time period	NA
Other analyses	17	Report other analyses done—eg analyses of subgroups and interactions, and sensitivity analyses	NA
**Discussion**			
Key results	18	Summarise key results with reference to study objectives	Page 10 line 1 – page 12 line 8
Limitations	19	Discuss limitations of the study, taking into account sources of potential bias or imprecision. Discuss both direction and magnitude of any potential bias	Page 11/ line 24-47
Interpretation	20	Give a cautious overall interpretation of results considering objectives, limitations, multiplicity of analyses, results from similar studies, and other relevant evidence	Page 12/ lines 1-8
Generalisability	21	Discuss the generalisability (external validity) of the study results	Page 11/lines 9-22
**Other information**			
Funding	22	Give the source of funding and the role of the funders for the present study and, if applicable, for the original study on which the present article is based	Page 13/lines 12-13

*Give information separately for exposed and unexposed groups.

Note: An Explanation and Elaboration article discusses each checklist item and gives methodological background and published examples of transparent reporting. The STROBE checklist is best used in conjunction with this article (freely available on the Web sites of PLoS Medicine at http://www.plosmedicine.org/, Annals of Internal Medicine at

For peer review only - http://bmjopen.bmj.com/site/about/guidelines.xhtml

http://www.annals.org/, and Epidemiology at http://www.epidem.com/). Information on the STROBE Initiative is available at www.strobe-statement.org.

For peer review only

For peer review only - http://bmjopen.bmj.com/site/about/guidelines.xhtml
